# Molecular Rationale behind the Differential Substrate Specificity of Bacterial RND Multi-Drug Transporters

**DOI:** 10.1038/s41598-017-08747-8

**Published:** 2017-08-14

**Authors:** Venkata Krishnan Ramaswamy, Attilio V. Vargiu, Giuliano Malloci, Jürg Dreier, Paolo Ruggerone

**Affiliations:** 1 Department of Physics, University of Cagliari, Cittadella Universitaria, S.P. Monserrato-Sestu km 0.700, I-09042 Monserrato, CA Italy; 2 Basilea Pharmaceutica International Ltd., Grenzacherstrasse 487, 4058 Basel, Switzerland

## Abstract

Resistance-Nodulation-cell Division (RND) transporters AcrB and AcrD of *Escherichia coli* expel a wide range of substrates out of the cell in conjunction with AcrA and TolC, contributing to the onset of bacterial multidrug resistance. Despite sharing an overall sequence identity of ~66% (similarity ~80%), these RND transporters feature distinct substrate specificity patterns whose underlying basis remains elusive. We performed exhaustive comparative analyses of the putative substrate binding pockets considering crystal structures, homology models and conformations extracted from multi-copy μs-long molecular dynamics simulations of both AcrB and AcrD. The impact of physicochemical and topographical properties (volume, shape, lipophilicity, electrostatic potential, hydration and distribution of multi-functional sites) within the pockets on their substrate specificities was quantitatively assessed. Differences in the lipophilic and electrostatic potentials among the pockets were identified. In particular, the deep pocket of AcrB showed the largest lipophilicity convincingly pointing out its possible role as a lipophilicity-based selectivity filter. Furthermore, we identified dynamic features (not inferable from sequence analysis or static structures) such as different flexibilities of specific protein loops that could potentially influence the substrate recognition and transport profile. Our findings can be valuable for drawing structure (dynamics)-activity relationship to be employed in drug design.

## Introduction

**Figure 1:**
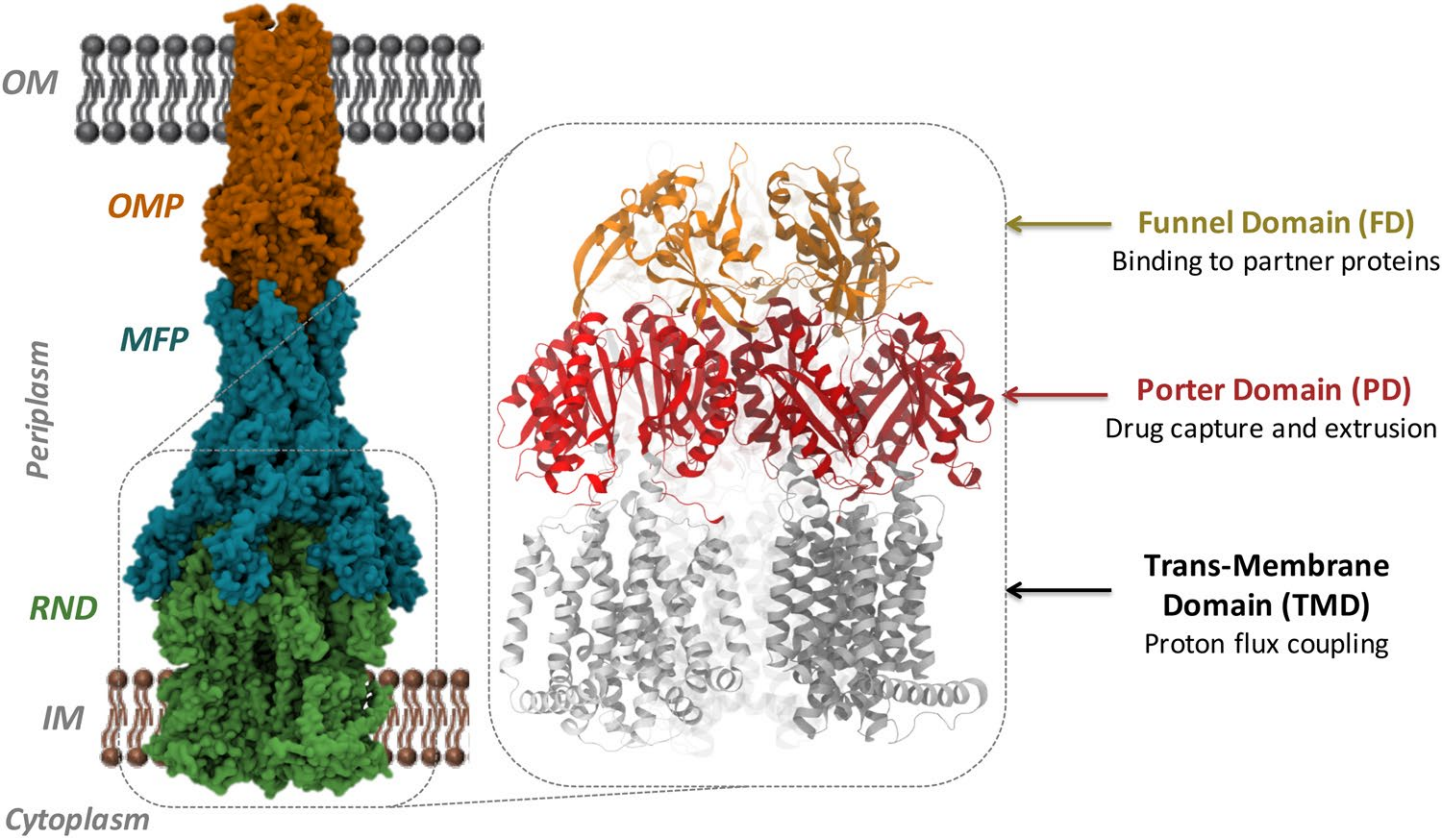
Structure of the RND tripartite pump AcrAB-TolC. AcrAB-TolC pump is shown here with AcrB as the RND component, AcrA as the MFP and TolC as the OMP. In the inset, the general structure of an RND transporter is displayed, where the three main domains (TMD, PD, and FD) are indicated.

Antimicrobial resistance has re-emerged as one of the major challenges to public health worldwide, especially due to the spread of multidrug-resistant (MDR) or even pan-resistant Gram-negative pathogenic bacteria^1^. The intrinsic drug resistance shown by these bacteria can be largely attributed to the primary barrier imposed by their membranes, endowed with chromosomally encoded molecular filters (porins) and drug efflux pumps^2^. Among these, MDR efflux pumps transport a wide range of structurally dissimilar substrates including antibiotics from various classes, posing a major concern in clinical therapy^3, 4^. In particular, the Resistance-Nodulation-cell Division (RND) superfamily members are notoriously known for the extremely wide substrate specificity^3, 5–8^ and are considered to be involved in both intrinsic and acquired MDR. The RND pump complexes span the entire periplasmic space from the inner membrane (IM) to the outer membrane (OM) by forming tripartite systems^9–13^ comprising an RND transporter protein embedded in the IM, an adaptor protein (a.k.a. membrane fusion protein, MFP) located in the periplasmic space, and an outer-membrane protein (OMP) constituting a long alpha-helical and beta-barrel tunnel (Fig. 1).

**Figure 2:**
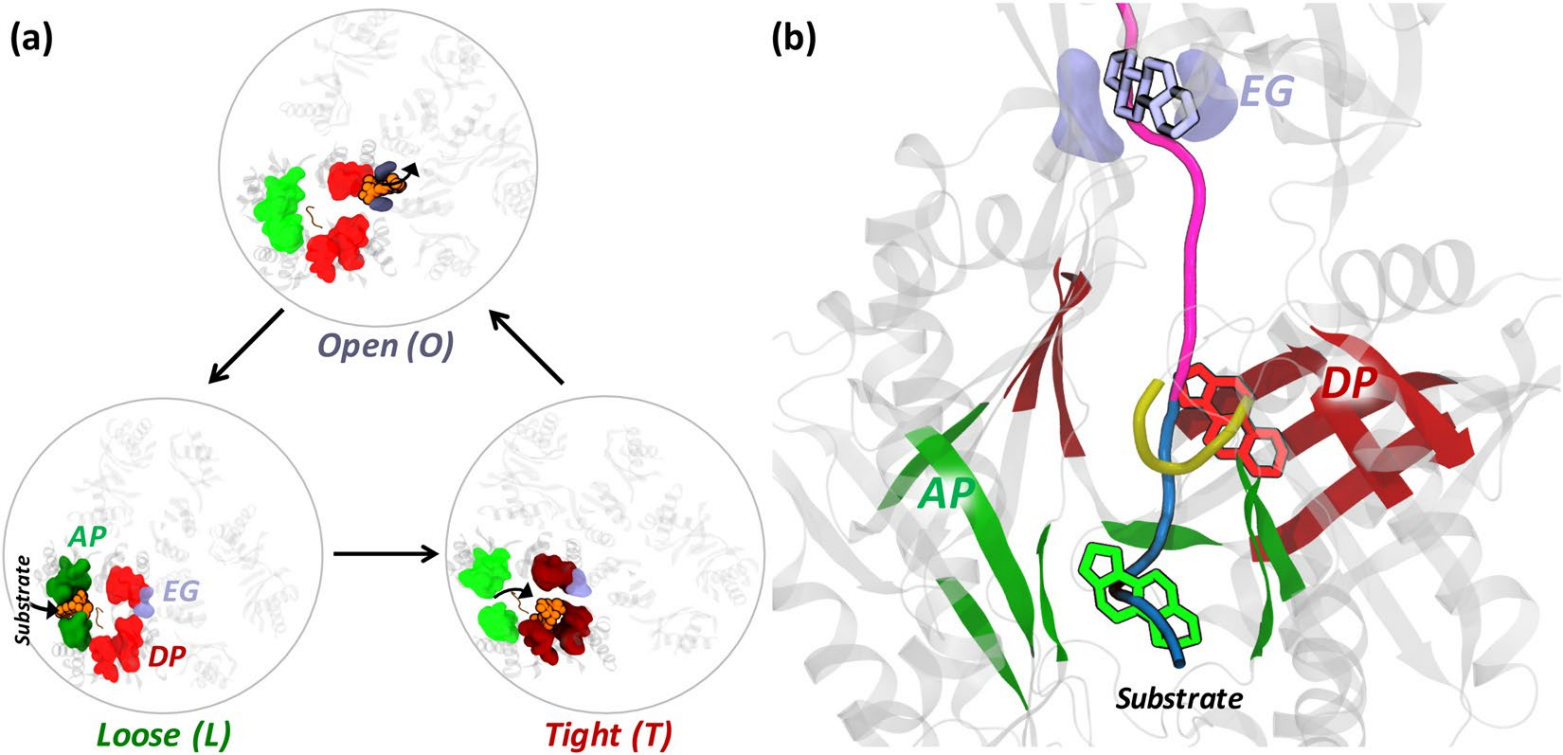
Proposed functional rotation mechanism of substrate extrusion by RND transporters. (**a**) Top view illustrating the different conformations assumed by AP, DP and Exit Gate (EG) during the three cycles of the functional rotation mechanism. The substrate (orange) is represented by van der Waals spheres and its pathway along the cycle is indicated by short black arrows. The key regions housing the substrate are coloured dull when binding to the substrate, bright otherwise. (**b**) Front view of the putative substrate transport pathway from AP to EG going through DP. The pathway is shown as thick tube, coloured in blue and magenta for the stages of the transport cycle associated to the *Loose*→*Tight* and *Tight*→*Open* conformational changes, respectively. The substrate is represented by sticks coloured green, red and iceblue when interacting with the AP, DP and EG (also coloured green, red and iceblue), respectively. The G-loop separating AP and DP is shown as a yellow cartoon.

AcrB is the best characterized RND transporter^8^, and its structure has been solved by several labs both without and with bound substrates and inhibitors^14–18^. Structurally, AcrB is an asymmetric trimer resembling a jellyfish with each protomer comprising a total of 3 domains^8^ (Fig. 1): (i) a trans-membrane domain consisting of 12 α-helices embedded in the IM, where energy conversion takes place via proton coupling; (ii) a pore (porter) domain located in the periplasm, where substrate recruitment and transport occur; and (iii) a periplasmic funnel domain, which connects the RND transporter to the OMP via the assembly of MFPs^19^ in the constituted pump. It has been proposed that substrate transport in these proteins follows a “functional rotation mechanism” (Fig. 2) in which concerted but not necessarily synchronous cycling of the protomers occurs through any of the so far identified asymmetric states: *Loose* (*L*) (a.k.a. *Access*) in which substrates bind to a peripheral site named access pocket (AP); *Tight* (*T*) (a.k.a. *Binding*) in which substrates bind to a more deep pocket (DP); and *Open* (*O*) (a.k.a. *Extrusion*) in which the substrate is released into the central funnel leading towards the OMP^14, 20, 21^. The two pockets (Fig. 2) were previously identified in AcrB as the binding sites responsible for the recognition and selectivity of different molecules^15, 22–24^. Namely, the AP and DP have been hypothesized to be responsible for the recognition of high-molecular-mass and low-molecular-mass compounds, respectively^15^. They are separated by a G-rich (a.k.a. switch) loop whose flexibility has been shown to be important for the transport of high-molecular-mass molecules^15, 16^.

AcrD is a close homolog of AcrB in *Escherichia coli* with an overall sequence identity (similarity) of nearly 66% (80%) (Supplementary Figs S1–S2). These moderate differences however impact on their substrate specificity patterns, which overlap only partially (Table 1 and Supplementary Fig. S3). While certain substrates like most of the beta-lactam antibiotics are common, macrolides and tetracyclines are transported by AcrB but not by AcrD, which instead exports aminoglycosides, in turn not recognized by AcrB. Categorizing the typical substrates of the two transporters on the basis of their physicochemical properties (Table 2) highlights that they are essentially hydrophobic and hydrophilic for AcrB and AcrD, respectively, while both transporters might shuttle out amphiphilic compounds.

**Table 1:** Antibiotic substrate specificities of the paralog RND transporters AcrB and AcrD from E. coli^22, 25, 46, 80–84^.

Transporter(s)	AcrB	AcrD	AcrB and AcrD
Antibiotic substrates	Macrolides (erythromycin), chloramphenicol, Fluoroquinolones (ciprofloxacin), Tetracyclines (tetracycline, tigecycline, minocycline), doxorubicin, acriflavine	Aminoglycosides (amikacin, gentamicin, kanamycin, neomycin)	Most beta-lactams (aztreonam, sulbenicillin), Aminocoumarins (novobiocin)

Classes of compounds are indicated, with examples of specific compounds within parentheses (2D chemical structures of these compounds are shown in Supplementary Figure S3).

**Table 2:** General physicochemical properties of antibiotic substrates of AcrB and AcrD.

Antibiotic substrate type (Physicochemical property)	*E*. *coli*	
	AcrB	AcrD
Hydrophobic	+	−
Hydrophilic	−	+
Amphiphilic	+	+

However, such a simplistic classification is not of help either to improve our basic knowledge on RND transporters or in drug design efforts. Achieving a deeper level of information would be highly desirable, and a first step towards this goal consists in mapping the differences in substrate specificities between these two proteins in terms of defined structural, chemical and dynamic features of their putative substrate-binding pockets, whose link has not been traced yet. From a domain-wise perspective, two previous studies attempted to identify substrate recognition site(s) in these RND pumps by using chimeric analysis^22, 25^. The importance of periplasmic loop regions in RND pumps was pointed out by Elkins and Nikaido^25^, Mao *et al*.^26^, Eda *et al*.^27^ and Kobayashi *et al*.^22^. In particular, Kobayashi *et al*.^22^ identified a few residues in the AP as potential determinants of specificity towards negatively charged beta-lactams (aztreonam, carbenicillin, and sulbenicillin). Namely, by replacing three residues in AcrB with the corresponding ones in AcrD (Q569R, I626R, and E673G), the authors were able to confer to the former transporter the ability to recognize anionic beta-lactams, typical substrates of the latter protein.

However, these findings concerning the overall location of the sites responsible for substrate recognition were restricted to a subclass of compounds, and no comprehensive molecular-level rationale for the different specificities of AcrB and AcrD has been proposed yet. This void of knowledge traces back mainly to the lack of experimental structures of AcrD and of co-crystal structures of any RND transporter with compounds belonging to beta-lactam, fluoroquinolone or aminoglycoside classes. On the other hand, computational modeling, in particular all-atom MD simulations, have already proven to be insightful in addressing the molecular mechanisms of RND transporters^8, 28–38^. Moreover, given the overall good sequence identity and similarity between AcrB and AcrD of *E*. *coli*, reliable computational modeling of AcrD and related structure-based studies are possible.

Prompted by this consideration and with the aim to explain in a more deep and informative meaning the substrate specificities of AcrB and AcrD in terms of matching properties with the corresponding (substrate) binders, we performed a systematic comparison of the physicochemical nature of the main putative substrate binding sites (AP and DP) (Fig. 2) between AcrB and AcrD. Importantly, beside crystal structures or homology models, we included for both transporters conformations extracted from extensive multi-copy μs-long molecular dynamics (MD) simulations. This robust computational setup allowed us to extend the realm of structure-function relation to account for subtle interplay between behavior of solvent, charge distribution, structural changes associated with the time-evolution of the system under physiological-like conditions. We characterized and compared the molecular properties (pocket descriptors) of the binding pockets such as their flexibility, accessible binding volume, lipophilic index, electrostatic potential, hydration and multi-functional sites. In particular, we identified dynamic features not inferable from simple sequence analysis, such as the positional flexibility of a loop lining the base of AP and likely playing a key role in regulating access and transport of substrates in these RND transporters. We also pinpointed specific differences in the lipophilic and electrostatic potentials in the binding pockets of these transporters, which complement the physicochemical properties of the known substrates of these pumps and are present also when dynamics of the pocket is accounted for. In particular, in AcrB an electrostatic funnel with negative gradient leading from the periplasm to the centre of the AP shows up in our configurations whilst it is absent in AcrD. Additionally, the DP of AcrB features a more lipophilic character, if compared to AcrD, pointing out the involvement of this pocket as a lipophilicity-based selectivity filter.

The correlation of the different specificity patterns of these two transporters to the dynamic physicochemical and topographical properties of their multi-functional recognition sites could be highly informative for drug design attempts^8^.

## Results

### Sequence Assessment

Bacteria have an inherent ability to change their genetic makeup and adapt themselves in response to adverse environmental stress. In order to extend the results of our study to Acr proteins extracted from *E*. *coli* other than the specific one used here, we determined the presence and distribution of conserved regions in AP and DP of all the available AcrB and AcrD sequences in the UniProtKB (October 2016) (http://www.uniprot.org/blast/). Shannon entropy^39^ was computed for both the Acr proteins and expressed in terms of H factor. This descriptor is commonly used to quantify sequence conservation considering the probability of occurrence of an amino acid at each site in a sequence alignment. Multiple sequence alignment and Shannon entropy analysis together pointed out an overall high sequence conservation of these proteins in all *E*. *coli* strains. In particular, all the H factors associated with the Shannon entropy were lower than 1, indicating a high degree of conservation.

### Molecular dynamics simulations of AcrB and AcrD

MD simulations of AcrB were done using the high-resolution asymmetric crystal structure (PDB ID: 4DX5^16^), each protomer being in *Loose*, *Tight*, and *Open* conformations, respectively. Since the structure of AcrD has not been experimentally resolved yet, we generated a homology model of AcrD based on the same AcrB crystal structure (4DX5) as its template using Modeller 9.13^40^ (see Methods section). According to RMSD analyses of the complete trimeric protein backbone and of each protomer in relation to the initial structure (Supplementary Fig. S4), we determined the equilibration time of ~0.5 μs to be most suitable for both AcrB and AcrD. The cluster representatives (see respectively Supplementary Figures S5 and S6 for the sampling of AP and DP clusters in AcrB and AcrD along the MD simulations) extracted from the equilibrated trajectories of AcrB and AcrD were used to characterize the distribution of accessible binding volume, molecular lipophilicity, electrostatic potential and multi-functional sites (MFS). Hydration analyses were performed on equilibrium trajectories. Although the level of confidence in homology models cannot be as high as that in experimental structures, we have thoroughly validated the AcrD structures by using state-of-the-art bioinformatic tools (details are reported in Methods and in Supplementary Information, see in particular Supplementary Table S1). The stability of the AcrD model as well as its suitability for subsequent analyses were validated in two independent μs-long MD simulations. This multiple validation of the AcrD model offers a fairly good confidence in the representativity of the clusters extracted from MD trajectories as configurations explored by the system. In the following, we present the results from these analyses on the AP and DP.

### Access Pocket of the *Loose* protomer

The AP is a proximally located pocket close to the periplasm in the putative substrate transport pathway of RND pumps (Fig. 2) and also likely the site of recognition for high-molecular-mass compounds^15^. In order to identify if any of the physicochemical properties could be used to differentiate between the APs and in addition to determine which substrate could likely be recognized by these APs, we calculated the following descriptors on the AP of *Loose* protomer in both AcrB and AcrD.

#### Pocket Volume and Shape

**Figure 3:**
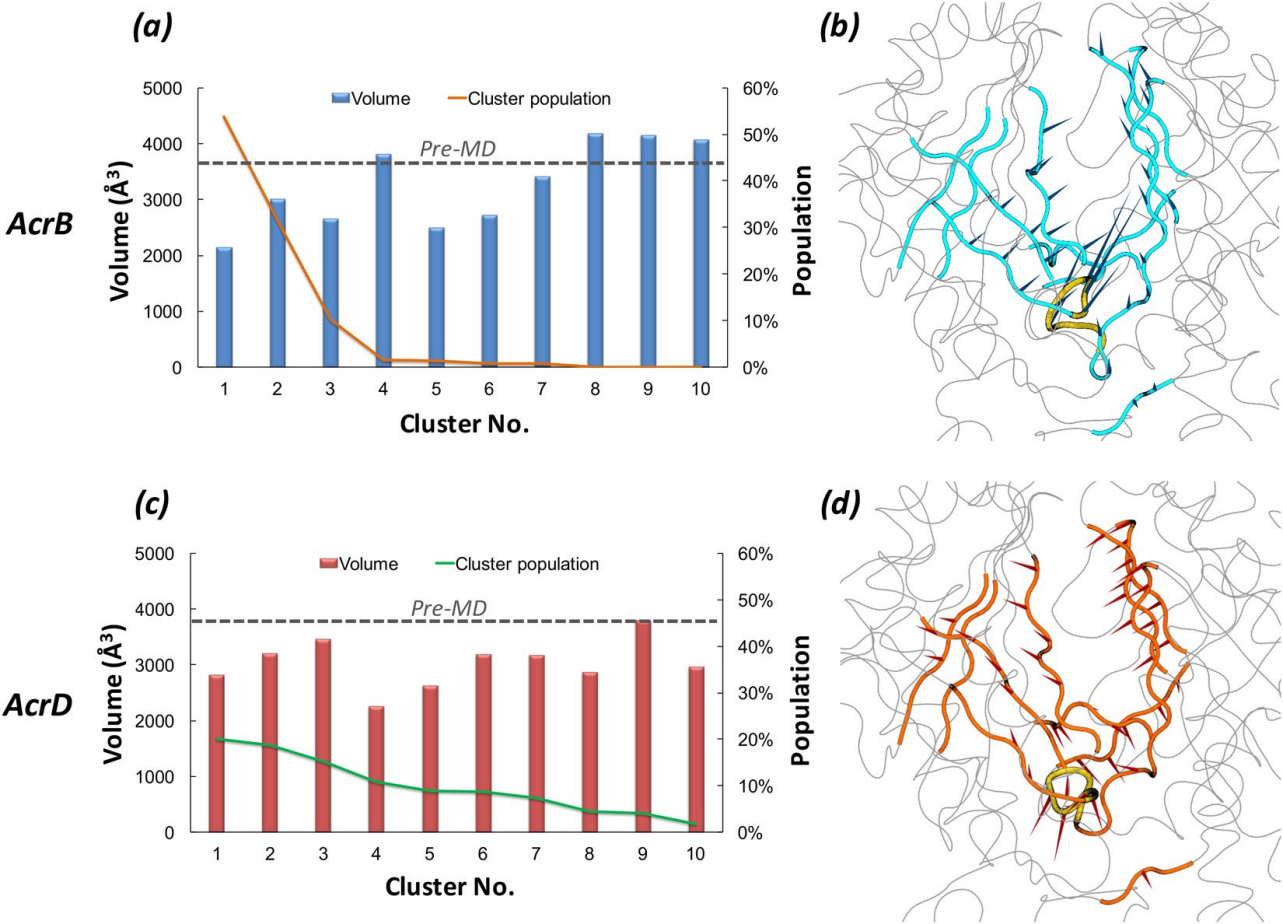
Volume dynamics of AP in the *Loose* protomer of AcrB and AcrD. (Left Panels) Distribution of the volume of AP within the *Loose* protomer of AcrB (**a**) and AcrD (**c**), calculated for the 10 top cluster representatives extracted from equilibrium MD trajectories. Histograms refer to the values of the volume, lines to the relative population of the corresponding clusters. The volumes calculated for the pre-MD structures of AcrB and AcrD are shown as dashed line. (Right Panels) Porcupine plots representing collective motions along the first principal-component eigenvector for AP in AcrB (**b**) and AcrD (**d**) simulations shown as arrows (>2 Å) attached to Cα atoms indicating the magnitude of the corresponding eigenvalues. The loop lining the base of AP (‘bottom-loop’) showing large rearrangement in AcrB but not in AcrD are coloured yellow in both.

The extent and the shape of the three dimensional space that a ligand is allowed to explore to find its optimal binding pose in any putative binding pocket is governed by multiple factors, the primary being the accessible binding volume. Especially with promiscuous proteins like the RND transporters, one would expect that a large binding site with a reasonable degree of plasticity will facilitate binding of molecules with a wide range of sizes. The pocket volumes (Fig. 3a and c and Supplementary Table S2) of pre-MD structures (PDB code 4DX5^16^ for AcrB and the final optimized model for AcrD used as starting configuration for MD simulations) and the clusters extracted from MD did not, per se, show any relevant differences. Moreover, both the volumes and the minimal projection areas of AP in AcrB and AcrD are much larger than those of the largest substrates transported by these pumps^41^ (Supplementary Table S2). However, principal component analysis performed on the equilibrium MD trajectories revealed a slightly different flexibility of the AP in the two proteins, namely the pocket in AcrB showed larger rearrangements in the loop residues 675 to 678 lining the base of AP (Fig. 3b) whereas in the case of AcrD (Fig. 3d) this region displayed lower flexibility and so was for the entire AP.

#### Molecular Lipophilicity Potential (MLP) and Lipophilic Index (LI)

**Figure 4:**
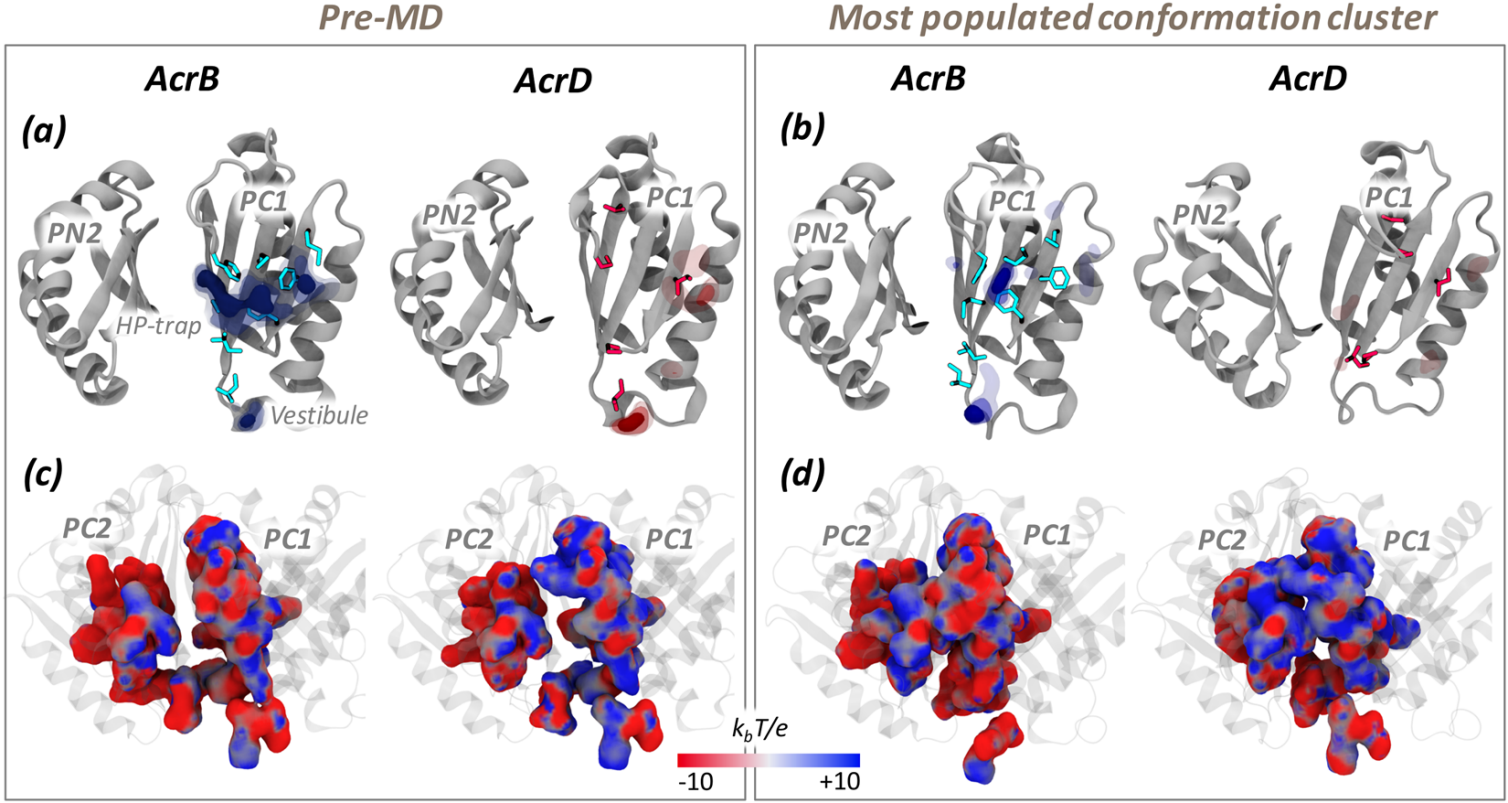
MLP and electrostatic potential of AP in the *Loose* protomer of AcrB and AcrD. MLP isosurfaces observed within 4 Å of AP in the *Loose* protomer of AcrB (blue) and AcrD (red) in pre-MD (**a**) and the representatives of the most populated cluster (**b**) as seen from the centre of the protomer. The hydrophobic/aromatic residues in the AP are shown as sticks in the structures. Isosurfaces at 0.75 (solid), 0.5 (dark transparent) and 0.25 (light transparent) are shown in blue (AcrB) or red (AcrD). The HP-trap and Vestibule sites are also labeled in the pre-MD structure of AcrB. The electrostatic potential plotted on the molecular surface representation of AP in the Acr proteins in the pre-MD (**c**) and the most populated cluster representative (**d**) as seen from the periplasmic front of the protomer. The colour code is red to blue from negative (−10 k_b_T/e) to positive (+10 k_b_T/e) potential, where k_b_ is the Boltzmann constant, T is the absolute temperature and e is the electron charge.

The calculation of the LIs of the AP showed this pocket to be of higher lipophilic nature in AcrB than in AcrD (Table 3 and Supplementary Fig. S7). This is compatible with the (at least partial) hydrophobic character required for AcrB substrates. However, the specific chemical environment of the AP is neither entirely hydrophobic nor entirely polar in both the proteins (Fig. 4a,b). Interestingly, different conformations of the AP displayed similar values of the LIs, so that the relatively higher lipophilicity of AcrB with respect to AcrD turned out to be a robust feature compared to the flexibility of the AP (Table 3). According to the MLP calculated for the representatives of the most populated structural clusters, regions of relatively high lipophilicity for AcrB were located close to the hydrophobic trap (HP-trap) lined by residues F136, F178, F610, F615 and F628^17, 29^, and in a region at the border with the putative entrance known as Vestibule^42^. In contrast, no predominant spots were recognizable for AcrD (Fig. 4a,b).

**Table 3:** Lipophilic indexes of AP in the *Loose* protomer of AcrB and AcrD.

System	LI	
	Pre-MD	MD clusters
AcrB	7.2	7.0 ± 1.0
AcrD	1.2	1.6 ± 0.6

For AcrB, the pre-MD structure corresponds to the crystal structure identified by PDB code 4DX5^16^ while for AcrD it is the final optimized homology model used as starting configuration for MD simulations.

#### Electrostatic Potential

The long range potential due to electrostatic interactions makes them a vital component of molecular recognition between molecules. The electrostatic potentials calculated on the molecular surfaces of the AP of AcrB and AcrD are shown in Fig. 4c,d. The left and right panels collect the results for the pre-MD structures and for the most populated cluster of each system, respectively. Concerning the pre-MD structures (Fig. 4c), positively charged patches were predominant within the AP of AcrD, while the same region of AcrB featured a more even distribution of positive vs. negative charges. Importantly, the partial closure of the pockets seen in the MD simulations of the apo proteins did not influence these main findings (Supplementary Fig. S8).

#### Hydration Analysis

**Figure 5:**
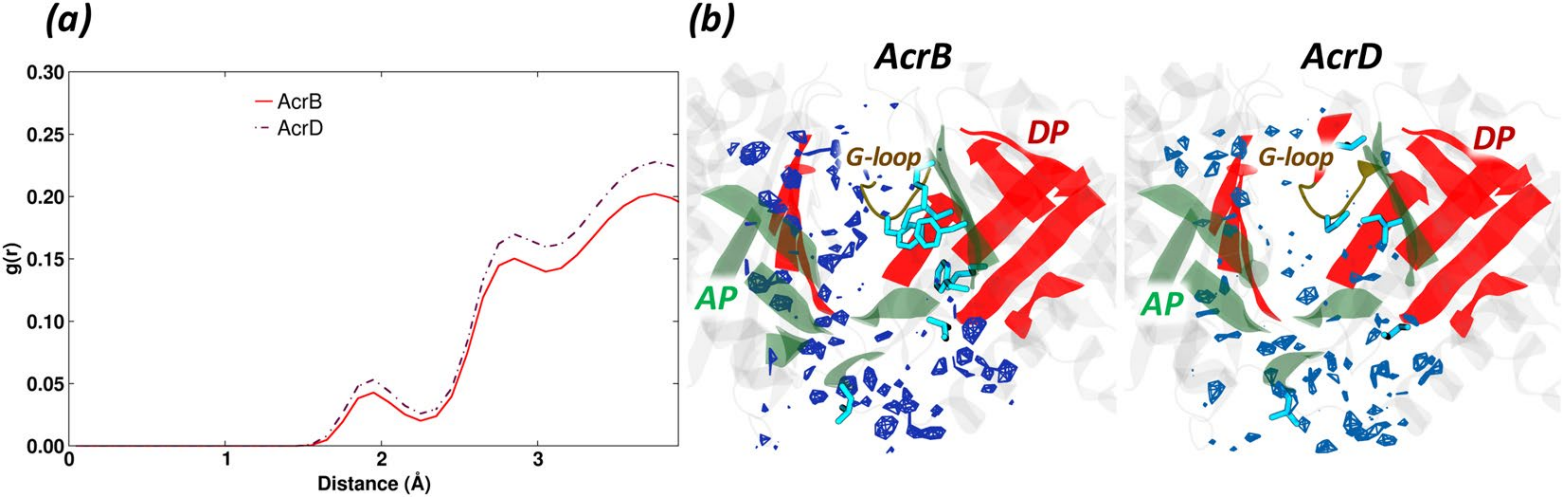
Hydration of AP in the *Loose* protomer of AcrB and AcrD. (**a**) Comparison of RDF profiles of water oxygen atoms around AP (all atoms) in the *Loose* protomer of AcrB (red solid line) and AcrD (brown dash-dotted line) extracted from the equilibrium MD trajectories. (**b**) Comparison of SDF of waters within the AP of *Loose* protomer. The SDF was calculated over the configurations forming the most populated cluster of AcrB (left) and AcrD (right). The isosurfaces are shown at density isovalue of 6, meaning that the represented surfaces correspond to 6 times higher average number density of solvent molecules than bulk (see Subsection Hydration in Methods). The AP and DP are marked in green and red while the G-loop in yellow cartoon representations. The hydrophobic/aromatic residues of the pocket are shown as cyan sticks in the respective structures.

Characterizing the hydration profiles around the binding pockets of these proteins helps to effectively understand the molecular mechanism of interaction of water molecules penetrating the pocket in a dynamic manner. The radial distribution function (RDF) profile around the AP residues of AcrB and AcrD were rather similar with only a minor difference in the intensity of hydration (Fig. 5a). The first solvation shell was observed around 1.9 Å in both the proteins with a slightly reduced probability in AcrB. The spatial distribution function (SDF) calculated on the trajectory of the most populated cluster extracted from MD simulations, however, featured no water density spots near the hydrophobic residues in AP of AcrB but showed a higher number of dense regions in AcrD at identical density isovalues (Fig. 5b).

### Deep Pocket of the *Tight* protomer

The DP is a more deeply located cavity within the putative substrate transport pathway of RND pumps (Fig. 2), and is likely the recognition site for low-molecular-mass compounds^15^. According to the crystal structures, this pocket exists in a collapsed state in the *Loose* and *Open* protomers but is wide open in the *Tight* protomer; therefore, all the analyses concerning this site were performed on the *Tight* protomers of AcrB and AcrD. Based on primary sequence analysis, most of the hydrophobic residues in the DP of AcrB are replaced by polar/charged amino acids in AcrD (Supplementary Figs S1–S2). The ensuing effects on the physicochemical environment of the DP were thus characterized by the aforementioned pocket descriptors.

#### Pocket Volume and Shape

**Figure 6:**
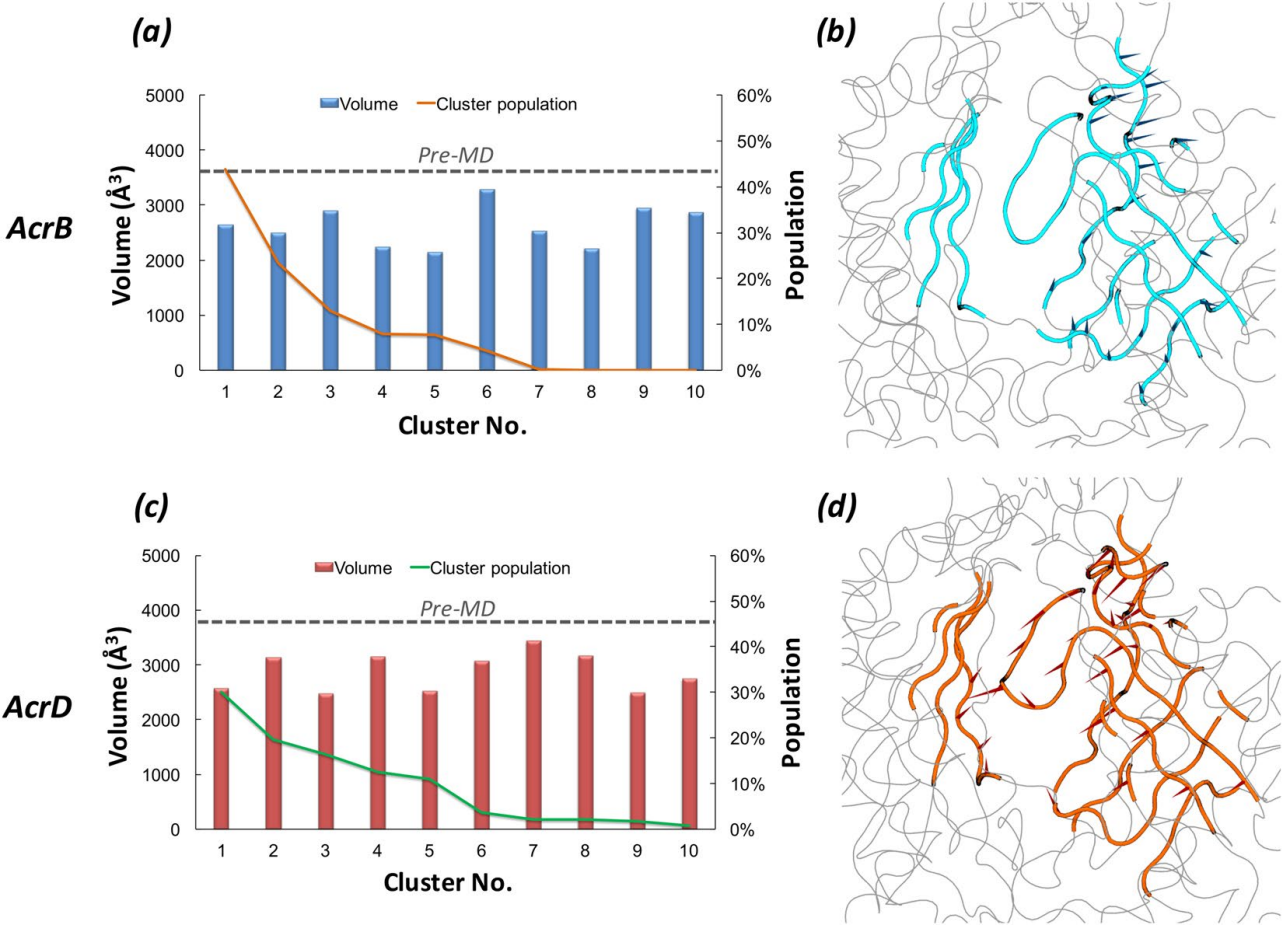
Volume dynamics of DP in the *Tight* protomer of AcrB and AcrD. (Left Panels) Volume distribution of DP in the *Tight* protomer of AcrB (**a**) and AcrD (**c**) over the simulation timescale. (Right Panels) Porcupine plots of the first principal-component eigenvector for DP in AcrB (**b**) and AcrD (**d**) simulations shown as arrows (>2 Å) attached to Cα atoms indicating the magnitude of the corresponding eigenvalues.

As for the AP, also the DP showed a partial closure during dynamics yet displaying volumes and minimal projection areas (Fig. 6a and c and Supplementary Table S3) large enough to accommodate its ligands. The cluster distribution was similarly slightly more extended for AcrD than for AcrB. The principal component analysis data showed the DP in AcrD with essential dynamics spread throughout the pocket unlike the less dynamic and more localized motions of DP in AcrB (Fig. 6b and d).

#### MLP and LI

**Figure 7:**
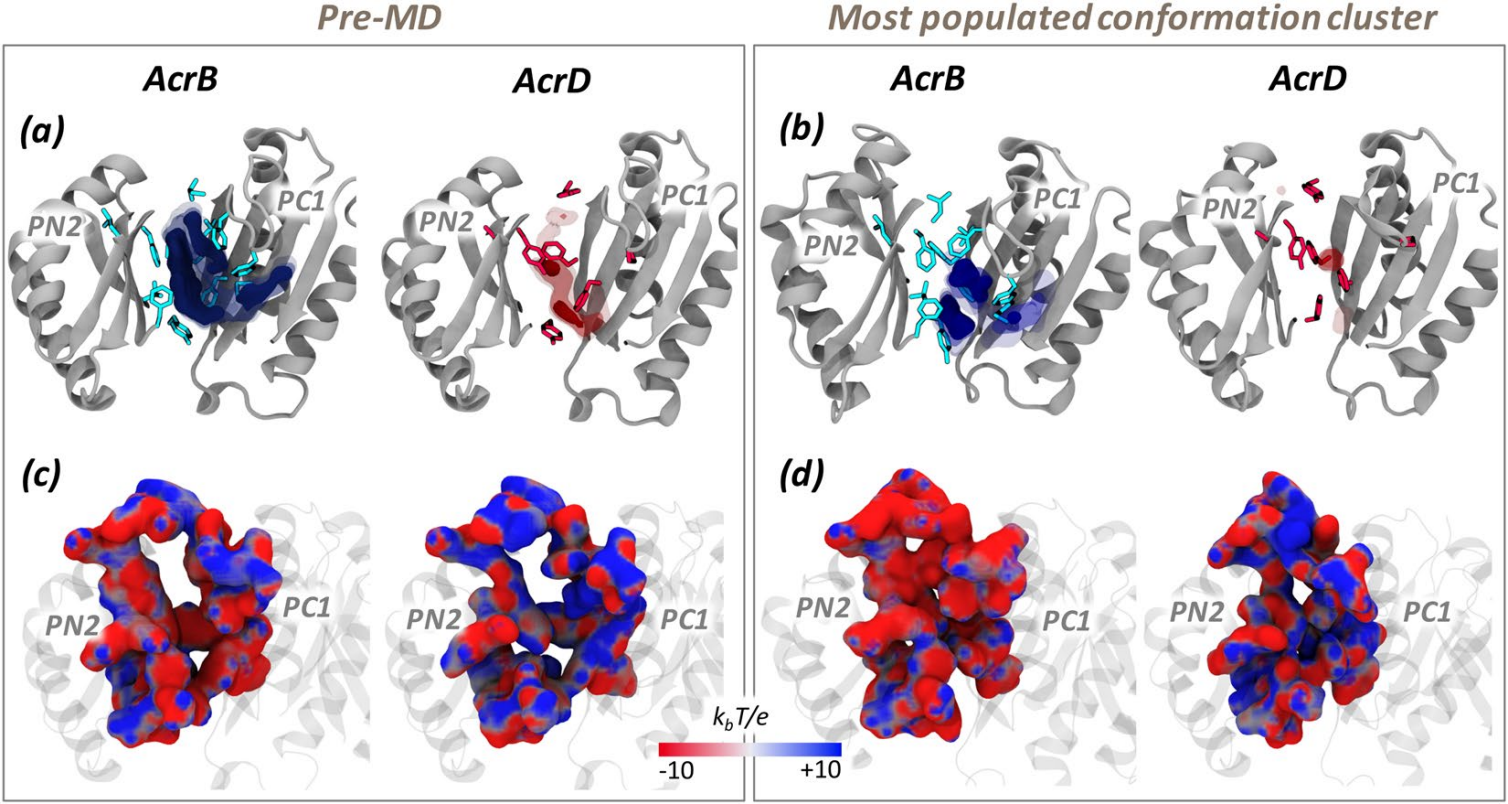
MLP and electrostatic potential of DP in the *Tight* protomer of AcrB and AcrD. See Fig. 4 for further details.

The DP featured larger differences than the AP in the values of the LIs calculated for AcrB and AcrD, despite a reduction in the absolute values when considering the weighted average value extracted from the MD clusters compared to pre-MD structures (Table 4). The LI values indicated a much more prominent hydrophobic character of this pocket in AcrB than AcrD. Most clusters of AcrB were associated with LI values larger than 10, the highest values occurring for clusters 1, 3, 5, and 8 (LI of 14.4, 17.1, 17.0, and 17.2 respectively; see Supplementary Fig. S9). Together, the first three clusters embraced 80% of the conformations sampled by AcrB. In AcrD, the most populated clusters (1 to 5) had LIs ranging from 0.5 to 2.8 (Supplementary Fig. S9). Therefore, as already seen for the AP, the MLP proved to be a robust feature of the pockets, conserved across different conformations assumed in the dynamics. The lipophilic potential surfaces of the pre-MD and the most populated clusters are reported in Fig. 7a,b highlighting the presence of pronounced lipophilic regions in AcrB in comparison to three less extended spots in AcrD.

**Table 4:** Lipophilic indexes of DP in the *Tight* protomer of AcrB and AcrD.

System	LI	
	Pre-MD	MD clusters
AcrB	16.3	13.1 ± 3.3
AcrD	4.9	1.9 ± 1.0

See Table 3 for further details.

#### Electrostatic Potential

The electrostatic potential projected on the surfaces of the DP indicated a relatively denser positive environment in AcrD than in AcrB (blue areas in Fig. 7c,d). Noticeable is that the difference was better emphasized when the electrostatic potential surfaces were compared for the representatives of the most populated clusters. In AcrB, an extended surface area of negative potential appeared while in AcrD the distribution of areas of negative and positive potentials did not change much and the latter still presented a greater positive component with dispersed negative components. As in the case of AP, the partial closure of the pockets seen in the MD simulations of the apo proteins did not influence these main findings (Supplementary Fig. S10).

#### Hydration Analysis

**Figure 8:**
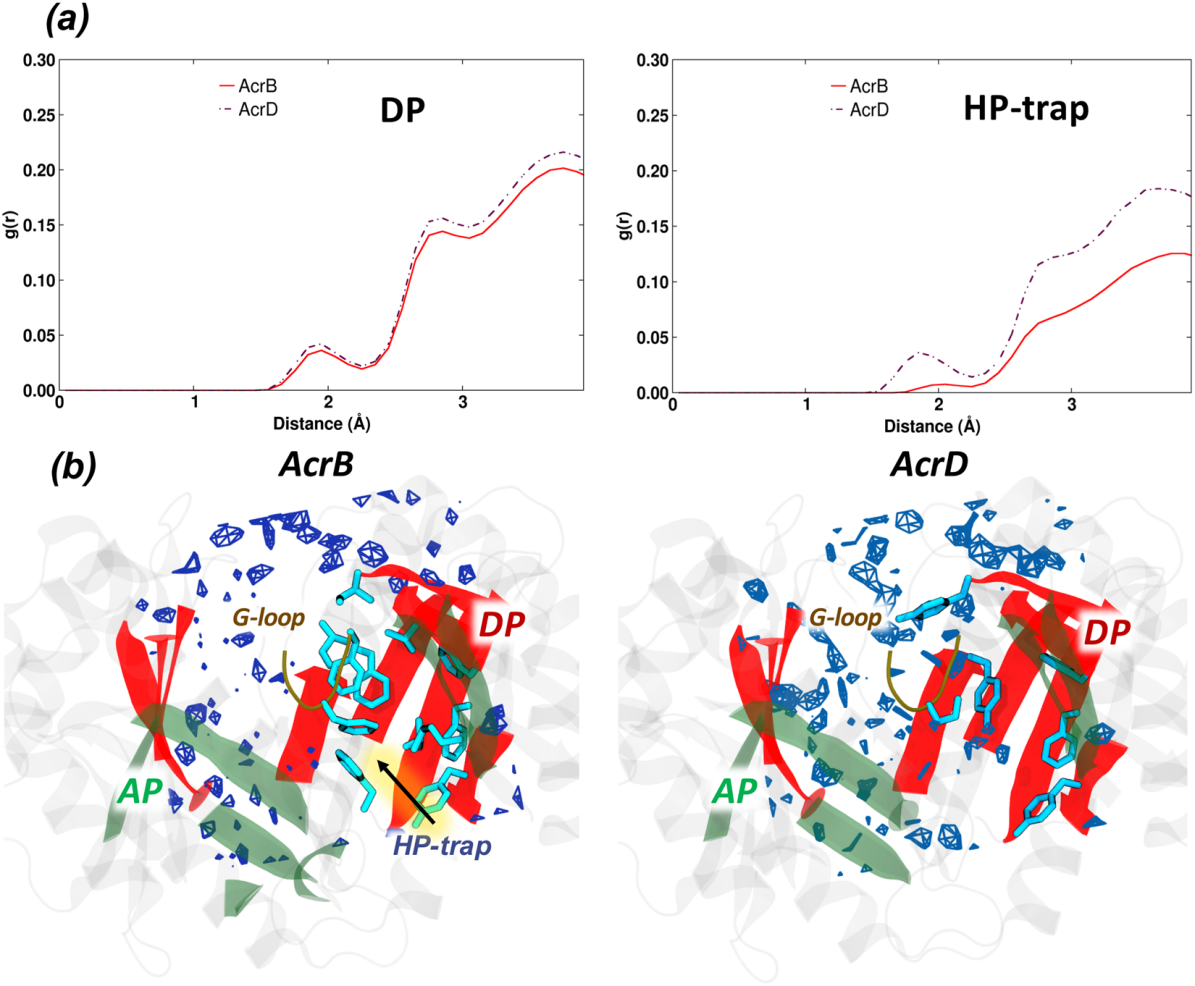
Hydration of DP and HP-trap in the *Tight* protomer of AcrB and AcrD. (**a**) Comparison of RDF profiles of water oxygen atoms around the DP (all atoms) and HP-trap (all atoms) in the *Tight* protomer of AcrB (red solid line) and the corresponding regions of AcrD (brown dash-dotted line). (**b**) Comparison of SDF for waters in the DP calculated over the configurations forming the most populated cluster of AcrB (left) and AcrD (right) illustrating the variation in the immediate environment of the hydrophobic residues. The position of the HP-trap in DP of AcrB is indicated by an arrow. See Fig. 5 for further details.

The RDF profile of water around the DP showed a minor difference in the intensity of the peak between AcrB and AcrD, essentially related to the different hydration of the HP-trap region^17^ (Fig. 8a). This is also consistent with the replacement of three out of the five phenylalanine residues in AcrD that are present in the HP-trap of AcrB (only F610 and F628 are conserved). Indeed, the SDF clearly displayed a very low probability of hydration near this region in AcrB (Fig. 8b).

### Fragment-Based Binding Site Characterization

In addition to global physicochemical pocket descriptors discussed above, the ligand-binding properties of protein are governed by the number, strength and spatial distribution of binding energy hot spots^43^. A fragment-based binding site analysis was thus performed employing probes (Supplementary Fig. S11) of different physicochemical features to identify hotspots responsible for specificity by mapping the chemical functionalities on the internal surface of the two proteins.

**Figure 9:**
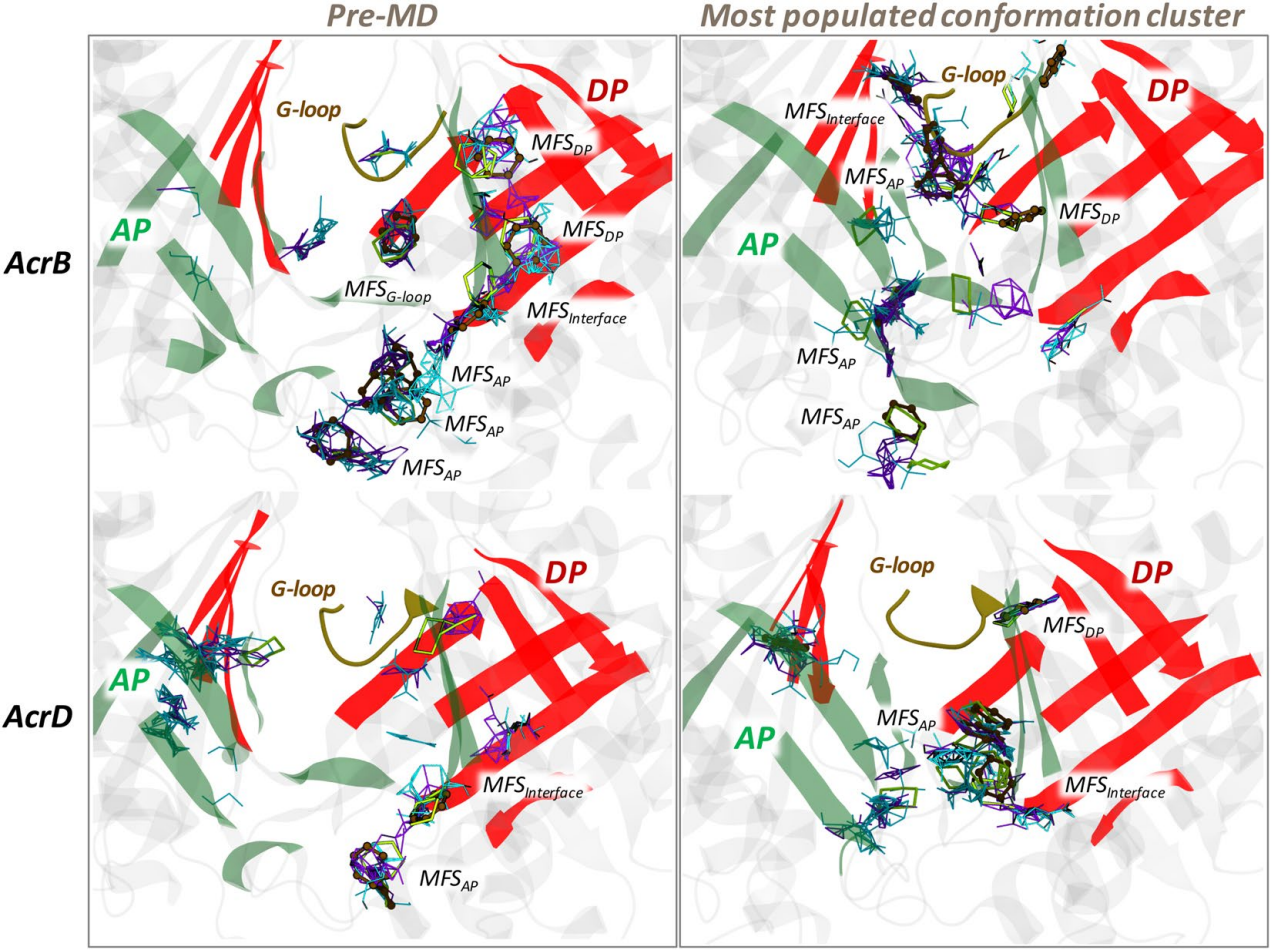
MFSs identified in the AP and DP of AcrB and AcrD. MFSs in the AP and DP of pre-MD (left panels) and the most populated cluster representative (right panel) structures of AcrB and AcrD. The binding modes of the different probes are shown as lines for hydrogen-bond donor (cyan), hydrogen-bond acceptor (violet) and aliphatic (yellow), and as CPK for aromatic (ochre) ligands. The AP and DP are marked in green and red, respectively, while the G-loop in yellow cartoon representations. (Note: The categorizing of MFSs here is arbitrary due to indistinct boundaries between the pockets. The sites not labelled as MFS here are all CSs).

Several multi-functional sites (MFSs) were identified within the binding pockets of AcrB and AcrD. While the AP of both proteins showed consistently higher number of MFSs in comparison to the DP (Table 5), the level of promiscuity became distinct on comparing the MFSs in the latter pocket. The DP in AcrB showed an extended MFS (Fig. 9) in the pre-MD structure where substrates like minocycline^14–16^, doxorubicin^14, 16^ and inhibitors like P9D^17^ and MBX2931^44^ were crystallographically resolved. Although closure of the DP during the simulations resulted in the loss of the large extended MFS found in pre-MD, it created other MFS thereby preserving the promiscuity of DP in AcrB as seen in the representatives of the most populated clusters (Fig. 9 and Supplementary Fig. S12). In AcrD, the DP and interface/G-loop showed only a few consensus sites (CSs) and lacked a true MFS in both the pre-MD as well as the clusters sampled during MD. An interesting feature was that the interface between the pockets including the G-loop almost always favored an MFS in AcrB.

**Table 5:** The number of MFSs identified in the binding pockets of AcrB and AcrD before and during MD.

Structure		Number of MFSs			Total number of MFSs
		AP	DP	Interface/G-loop	
AcrB	Pre-MD	3	2	2	7
	Cluster 1	3	1	1	5
	Cluster 2	2	1	1	4
	Cluster 3	1	2	1	4
	Cluster 4	2	1	1	4
	Cluster 5	2	1	1	4
	**Average**	**2**.**2**	**1**.**3**	**1**.**2**	**5**
AcrD	Pre-MD	1	-	1	2
	Cluster 1	1	1	1	3
	Cluster 2	3	-	-	3
	Cluster 3	3	-	1	4
	Cluster 4	3	-	-	3
	Cluster 5	3	1	-	4
	**Average**	**2**.**3**	**0**.**3**	**0**.**5**	**3**

See Table 3 for further details.

## Discussion

AcrB and AcrD are the major RND transporters of *E*. *coli*. They feature an overall good level of sequence (and likely fold) identity and similarity, and indeed show partly overlapping substrate specificities. However, they have distinct abilities to expel some classes of compounds; for instance, only AcrD recognizes aminoglycosides. The peculiarities of each transporter are likely related to the specific physicochemical features of the main recognition pockets, i.e. the AP and DP. Indeed, these two sites feature a lower degree of sequence conservation compared to the entire protein (see Supplementary Figures S1–S2). In particular, the AP is better conserved than the DP with nearly 60% vs. 40% identical residues, respectively. An inspection of the mismatched residues between AcrB and AcrD showed that the binding pockets of the latter protein are populated with more polar/charged residues than those of the former, likely facilitating the recognition and transport of more hydrophilic molecules by AcrD. However, this hypothesis should undergo a validation through the rationalization of the different substrate susceptibilities in terms of molecular descriptors of the binding pockets. Moreover, the impact of the dynamic nature of these transporters on the pocket environment should also be considered for gaining a more realistic understanding of their differential recognition and transport events as seen *in vitro* or *in vivo*.

For this reason, in this work we compared several molecular descriptors calculated on the two main putative binding sites (AP in the *Loose* protomer and DP in the *Tight* protomer) within the periplasmic domains of AcrB and AcrD. In addition to experimental structures and homology models, which represent static snapshots of these biologically dynamic systems, we performed our analyses on a set of structures extracted from extensive MD simulations of the apo-proteins for assessing the influence of pocket dynamics. We recall that the MD simulations of AcrD were started from a homology model built using the AcrB structure as template. Clearly, the structures of cluster representatives for the former protein could feature a lower level of confidence than those of the latter. However, the AcrD model was found to be as good as experimental structures using state-of-the-art bioinformatic validation protocols (see Methods). In addition, the MD simulations of AcrD were as stable as those of AcrB, further pointing to the reliability of our findings.

**Figure 10:**
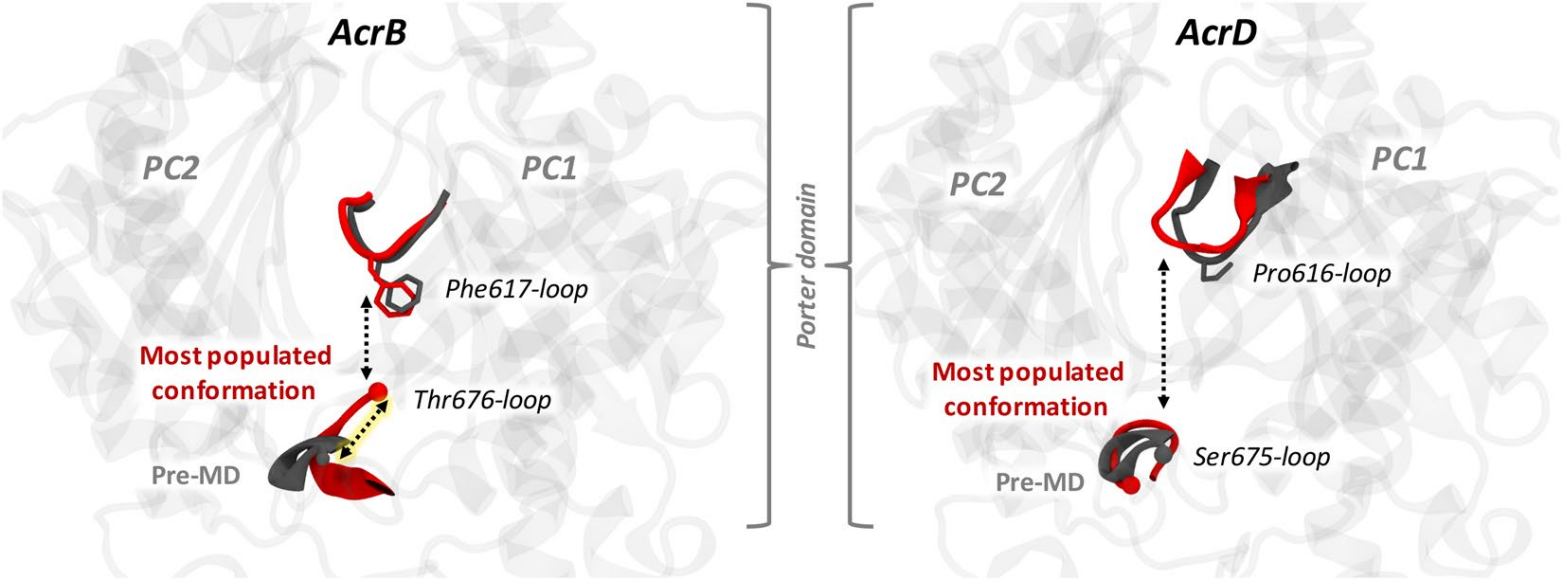
Main conformational states of the Thr676-loop (bottom-loop) in the *Loose* protomer of AcrB (left panel) and of the corresponding Ser675-loop in AcrD (right panel). The conformation of the most populated clusters and the pre-MD structures are shown in red and grey cartoons, respectively. The conformations of the G-loop are also indicated with the same colour code.

The first descriptor we considered was the pocket volume and shape. However, a pure steric filter for substrates is quite unlikely because of the large volumes of all the pockets considered in the present analysis, which are at least twice as voluminous as the largest compounds transported by AcrB and AcrD. Moreover, the average values of the volumes and minimal projection areas for the AP and the DP of both proteins are, within errors, very similar; therefore, differences in the substrate specificities of AcrB and AcrD cannot be traced back to the size of such large pockets. Interestingly, in both transporters the two pockets partly collapsed during the MD simulations with respect to the conformation seen in the X-ray crystal structures and in the homology models of AcrB and AcrD, respectively. The reduction amounted to 30% in both AP and DP of AcrB while it was 15% in the AP and 28% in the DP of AcrD with respect to the pre-MD structures. This behavior is consistent with the findings of Fischer and Kandt^33^, who noticed a closure of the DP in the *Tight* protomer of AcrB in the absence of substrate during shorter MD simulations than those reported here. In coherence with this hypothesis, we also found the DP volume to be at least 1000 Å^3^ larger in substrate bound complexes (Supplementary Table S4). Moreover, population distributions over the clusters extracted from the MD simulations offer interesting insights into the different behavior of the two transporters. First, for both AP and DP, the first three clusters identified for AcrB cover roughly 90% and 80% of the trajectories whilst the distribution is wider for AcrD, especially when AP is considered. Straightforwardly attributing this diversity to dissimilar flexibility might not be completely correct and could hide interesting features associated with the dynamics of the considered regions. For instance, as visualized from the porcupine plots of the first principal component (Fig. 3b and d), the entire AP of AcrD exhibits almost a coherent motion with similar magnitude of eigenvector (depicted by length of the arrows) whereas in the case of AcrB, the loop residues 675 to 678 lining the base of AP show larger rearrangements. The dynamicity of this loop (Thr676-loop or hereafter referred to as ‘bottom-loop’) represents a peculiar feature in the AP of AcrB, which is unshared with AcrD. The structures of the cluster representatives of AcrB can be partitioned in two groups featuring “up” and “down” conformation of the bottom-loop (Fig. 10). The most populated cluster is characterized by an “up” conformation, while the crystal structures exhibited only “down” configuration, as for the second most populated cluster representative (Supplementary Fig. S13). A similar flip is not observed in AP of AcrD, and the analogous of the bottom-loop is always close to the pre-MD arrangement. With such major conformational shifts of the bottom-loop in AcrB, it is very likely that this loop contributes towards induced fit and minimizes the steric hindrance for the large substrates of AcrB, a hypothesis that is compatible with the larger size of some AcrB substrates that are not transported by AcrD. The importance of this bottom-loop in regulating access to porter domain and its possible active role in substrate transport by pushing compounds towards the hydrophobic binding pocket was already suggested by Fischer and Kandt, who however sampled only “down” conformations in their MD simulations^33^. Moreover, according to Kobayashi and co-workers the mutation E673G (located close to the bottom-loop) in AcrB, in combination with Q569R and I626R, conferred this protein the ability to recognize anionic beta-lactams, which are typical substrates of AcrD. Thus, the analysis of the volumes, although not much enlightening per se, allowed identifying specific structural features more directly involved in the entrance into and transport to a pocket, which might be of relevance in determining substrate specificity.

Next to the volume analysis, we calculated the LIs for the AP and the DP of both transporters in order to quantify how different distribution of hydrophobic residues could affect substrate recognition and how this property is tuned by the dynamics of the protein. Both pockets of AcrB are consistently characterized by higher LI values than those of AcrD, independently of the somewhat important structural changes occurring in these pockets during the MD simulations of the apo-proteins (Tables 3 and 4). The higher lipophilic nature of AP and DP in AcrB when compared to that of the same pockets in AcrD is required for AcrB to provide a favorable environment for its hydrophobic substrates to bind. However, the specific chemical environment of the AP is neither entirely hydrophobic nor entirely polar in both the proteins. Such a dispersed nature binds ligands with different physicochemical properties by weak polar and hydrophobic interactions^45^, while facilitating easy transport by preventing strong interactions of the substrates with residues of the pocket. Note that the values of the LIs for AP and DP of AcrD are essentially identical whilst in AcrB there is a marked difference between the two sites, the DP being the more lipophilic. This could be an indication of DP being the site where substrates might be differentiated between AcrB and AcrD in terms of their lipophilicity. In other words, the DP could function as a lipophilicity-based selectivity filter for low-molecular-mass compounds. This proposal agrees with previous suggestions based on experimental results of Yamaguchi *et al*.^46^. The difference between the DP of AcrB and AcrD became even more prominent by comparing their molecular lipophilic surfaces (Fig. 7a,b). The MLP isosurfaces are significantly wider in AcrB than in AcrD, which correlates well with the nature of the reported substrates transported by the former protein. Interestingly, the presence of phenylalanines in the G-loop of only AcrB creates a large hydrophobic bridge between the DP and the AP, which would facilitate anchoring of aromatic compounds from the AP and their subsequent transport to the DP. The presence of polar/charged residues in the DP of AcrD results in its increased hydration when compared to the DP of AcrB, and the nature of water dynamics in this region would further influence the binding behavior of potential substrate molecules.

The local stereochemistry and distribution of functional groups in a region govern both the ordering of water molecules and their biologically important interactions in that region. The structure and the dynamics of the first water hydration shells around a putative binding pocket is of primary importance, given the relevance of water displacement for the free energy balance of the recognition event^47^. The plot of the SDF around the AP and the DP of both transporters highlight how their different hydrophobic potentials influence hydration profiles. In particular, a lower degree of hydration was seen near the hydrophobic part of the AP in AcrB than in AcrD (Fig. 5b). Even for the DP, our analysis provides a clear evidence of the contribution of the HP-trap in determining the lower hydration of the domain compared to AcrD. While several spots are homogeneously distributed in the SDF calculated for AcrD, the DP featured several zones without hydration in AcrB, especially around the HP-trap (Fig. 8b).

**Figure 11:**
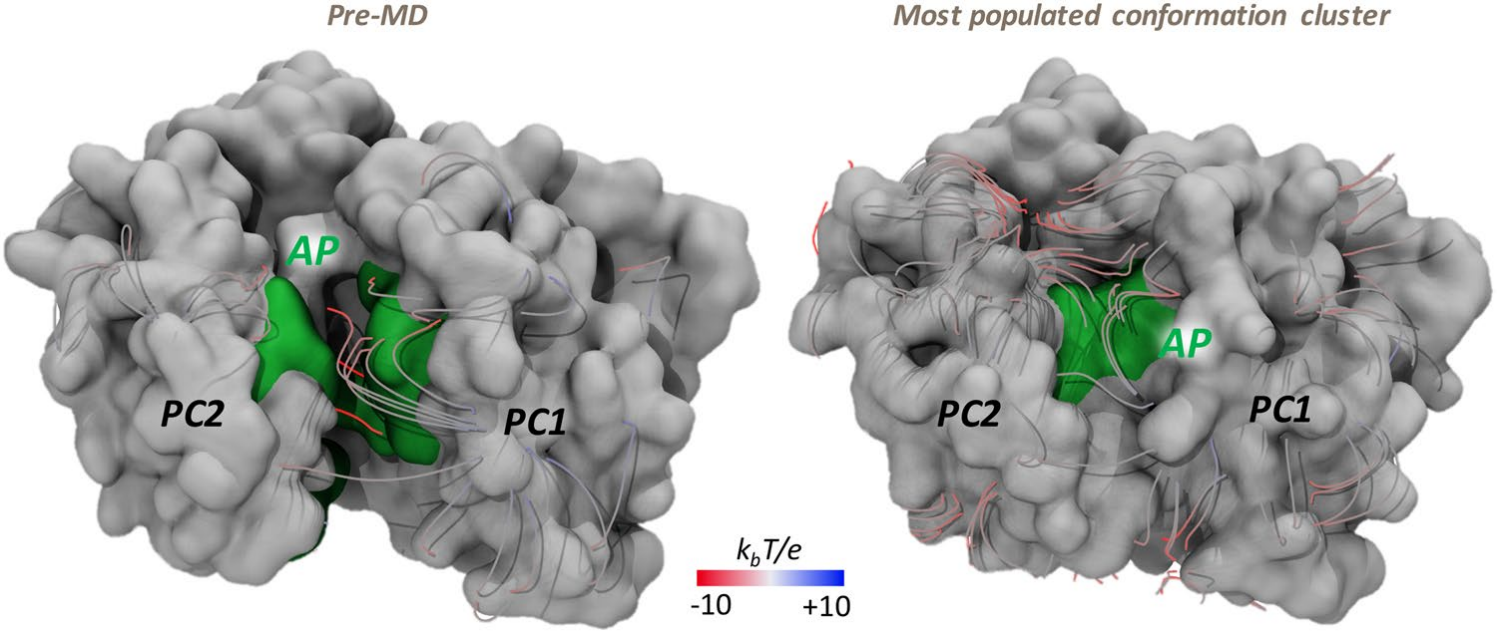
Electrostatic funnel converging into AP in the *Loose* protomer of AcrB. Only the strongest field lines are shown and coloured red to blue from negative (−10 k_b_T/e) to positive (+10 k_b_T/e) potential (‘k_b_’ is the Boltzmann constant, ‘T’ is absolute temperature and ‘e’ is charge of an electron). AP is shown in green and the rest of the porter domain in white surface representation.

Electrostatic complementarity between the pocket and substrate molecules is essential for initial substrate recruitment and augmentation of their association rate^48^. Therefore, this analysis is of particular interest for the AP, which is more peripheral than the DP. For AcrB, the distribution of positively and negatively charged patches on the molecular surface of this site is fairly homogeneous (Fig. 4c,d). Interestingly, in AcrB an electrostatic funnel with negative gradient leading from the periplasm to the centre of the AP is recognizable in Fig. 11, which could help the long-range recognition of positively charged compounds, and is compatible with the monocationic character of several substrates of AcrB. The electrostatic surface of this site in AcrD reveals instead a marked positive patch on the upper part, also due to the presence of residues like Arg568 and Arg625, which have been recently reported as key residues for specificity of AcrD towards negatively charged molecules like the anionic beta-lactams^22^.

Concerning the DP, the large electrostatic negative environment presented to the incoming substrate in AcrB would favor the recognition and binding of cationic compounds. However, this becomes unfavorable to hydrophilic polycationic aminoglycosides due to the high lipophilicity of this pocket in AcrB functioning as a probable lipophilicity-based selectivity filter as discussed above. In AcrD, a relatively denser positive environment compared to AcrB is identifiable, originating from the electrostatic contributions of amino acids like Arg and Lys replacing their less polar counterparts in AcrB (Supplementary Figs S1–S2). In conjunction with the low lipophilicity of this pocket in AcrD, the observed mosaic-like electrostatic patches provide a favorable binding site for anionic beta-lactams as well as for polycationic aminoglycosides. On the other side, the poor electrostatic and hydrophilic complementarity provided by the DP in AcrB permits the binding of charged molecules like anionic beta-lactams but with far less affinity than that in AcrD.

Multidrug transporters are known to have large, flexible overlapping substrate binding pockets rich in polar and aromatic residues to bind substrate molecules at different locations with different orientations. In alternate terms, these proteins show polyspecificity with no inherent ligand specificity which otherwise could stem from the binding site geometry^49^. Our results suggest that features such as shape, lipophilicity, electrostatic potential and hydration of AP and DP are a few distinctive features between AcrB and AcrD. This is in agreement with the findings on other multidrug transporters where nonpolar and aromatic side chains impose specific prerequisites on drug size and shape. To further establish this, we performed a fragment based binding site characterization using FTMap, whose philosophy retraces experimental high-throughput and fragment screening methods. Also, as its algorithm does not rely on alternate measures of ligand-binding propensity such as pocket volume, cavity depth or the ability of binding non-polar spheres, the results obtained here can complement those from physicochemical pocket descriptors discussed above.

As evident from the overall distribution of MFSs (Table 5), both AcrB and AcrD provide multiple binding possibilities with different functionalities as expected from such promiscuous transporter proteins. This is distinct from the limited number of MFSs one would detect in restrained substrate binding sites of other ordinary substrate-receptor systems restricted to a specific class of substrates^45^. In particular, AcrB with its numerous and wide spread MFSs offers a greater level of promiscuity for diverse substrate types than AcrD, which with its smaller localized MFSs puts forth certain prerequisites on the substrates being recognized. While the AP in both proteins showed comparable numbers of MFSs dispersed within the pocket, a clear distinction is noticeable in the DP (as also observed with the pocket descriptors discussed above) where a true and wide spread MFS is seen only in AcrB. The DP of this transporter is clearly a multi-functional (or multidrug binding) site with higher preference towards hydrophobic and aromatic fragments alongside hydrogen-bond donor and acceptor fragments. The non-selective characteristics of weakly polar (by Q176, G616) and weakly hydrophobic (by F178, Y327, F615, F617, F628) interactions are a predominant player towards the promiscuous binding behavior of AcrB DP^30^. The DP of AcrD does not reach the level of multi-functionality seen in AcrB, and shows greater preference for hydrogen-bond donors/acceptors (by R44, D134, N136, T139, Y178, Y327, S614, G615), together with a very limited preference towards hydrophobic (by Y178, Y277, F627) fragments. The MFSs identified here are in good agreement with the data reported for AcrB by Imai *et al*.^45^, and were also found close to the residues identified as crucial for the recognition of anionic beta-lactams by Kobayashi *et al*.^22^, thereby strengthening the reliability of our findings.

As seen from the distribution of MFSs in the various MD clusters (Supplementary Fig. S12), their position is not constant and this dynamicity (attributed by spatial changes in internal cavities caused by peristaltic motions^50^) is most likely important to avoid the substrate from being trapped in a single site and to facilitate its efflux by multisite-drug-oscillation^46^. Additional studies involving substrate bound complexes can provide information on the interaction profile of these homologous Acr pumps with their corresponding substrates. However, redundancy in the residue type in the binding pocket leading to easy adaptability of the binding orientation of substrates in the presence of mutations as identified by Bohnert *et al*. in AcrB^51^ makes these pumps very challenging for studies with simple molecular docking^30^.

## Conclusions and Perspectives

In this study, we performed a comparative analysis of the physicochemical properties like pocket volume and shape, lipophilicity, electrostatic potential, hydration and multi-functional sites of AcrB and AcrD to rationalize their differential substrate specificities. Importantly, these analyses were performed not only on static structures but also on conformations extracted from extensive MD simulations accounting for the impact of protein dynamics. Our results reveal several features in which both the AP and the DP differ considerably between these two transporters. First, the calculated lipophilic potential turned out to be significantly different between the AP and DP of AcrB and between the corresponding pockets of AcrB and AcrD considering even the dynamics of the pockets. In particular, the DP of AcrB is more lipophilic than all other sites, suggesting the possible role of this pocket as a lipophilicity-based selectivity filter. Second, we observed specific differences in the electrostatic environment within the pockets. In particular, the presence of an electrostatic funnel sourcing from the AP of AcrB could be important for the recognition of monocationic compounds by this transporter. Thus, these two properties likely play a central role in governing substrate recognition by and specificity of AcrB and AcrD. Meanwhile, the cavity volume, which essentially remains large enough to accommodate all potential substrate molecules, possibly has an indirect effect on the lipophilic and electrostatic environment along with the distribution of MFS, which altogether with the ensuing hydration within the pocket govern recognition and transport of substrates by these pumps. In addition, specific features like the flip conformations of the bottom-loop and the lipophilic bridge created by Phe617 of G-loop both in AcrB (and not in AcrD), which could not have been identified from simple sequence analysis, are expected to play a key role in the recognition and transport function of these pumps.

More exhaustive studies including molecular docking and molecular dynamics simulations of selected substrates in the binding pockets of AcrB and AcrD are being considered to provide substantial information to further characterize these putative binding sites on the basis of substrate-protein interaction pattern.

## Methods

### Homology modeling of AcrD

A reliable structure of the system of interest is the starting and main ingredient of any structure-based computational study. Since the structure of AcrD has not yet been resolved experimentally, we built it by template-based homology modeling. The amino acid sequence of full length AcrD transporter protein from *E*. *coli* was retrieved from the UniProt database^52^ (UNIPROT ID: P24177), and subsequently searched for the best available template structures bearing homologous relationship to the query sequence using the NCBI-BLAST tool^53^ against the Protein Data Bank (PDB) (www.rcsb.org). AcrB sequence showed the highest identity (~66%) (similarity of ~80%) with least gaps over a maximum sequence coverage; therefore its high resolution crystal structure, 1.9 Å (PDB ID: 4DX5^16^), was chosen as template for modeling AcrD. The two protein sequences were optimally aligned by ClustalOmega^54^ and the results were visually inspected to ensure the absence of gaps in important secondary structure regions. Modeller 9.13^40^ was used to generate a total of 100 asymmetric models of AcrD based on AcrB template using an optimization method combining slow MD with very thorough variable target function method through 300 iterations, and this whole cycle was repeated twice unless the objective function MOLPDF was greater than 10^6^. The resulting models were ranked using discrete optimized protein energy (DOPE)^55^ score values, and the top 5 models (with the lowest DOPE score) were selected for individual structure quality checks. Each model was further subjected to loop refinement using Modeller, and to overall structure relaxation by energy minimizations using AMBER14^56^. The most reliable model was then selected based on various geometric and stereochemical quality factors evaluated for backbone angles, side chains flips, rotamers, steric clashes etc. using PROCHECK^57^, ERRAT^58^, ProSA^59^, Verify3D^60^ programs available in MolProbity^61^ and Structure Analysis and Verification Server (http://services.mbi.ucla.edu/SAVES/).

We also performed comparative structural studies by superimposition of the modeled AcrD structure over the experimentally determined X-ray crystal structure of AcrB used as the template. All the above methods were also employed on the crystal structure of AcrB for use as reference. Visual inspections were performed with VMD1.9.1^62^ and PyMOL^63^.

### Molecular dynamics simulations of AcrB and AcrD

MD simulations of the crystal structure of AcrB (PDB ID: 4DX5) and of the most reliable homology model of AcrD (see Supplementary Table S1) were carried out using the AMBER14 molecular modeling software^56^. Protomer specific protonation states^18^ were adopted with E346 (E346) and D924 (D922) protonated in both *Loose* and *Tight* protomers while deprotonated in the *Open* protomer of AcrB (AcrD). The residues D407 (D407), D408 (D408), D566 were protonated only in the *Open* protomer of AcrB (AcrD). The charge state of the residue L565 of AcrD, corresponding to D566 in AcrB, was not modified of course. The topology and the initial coordinate files for these apo-protein structures were created using the *LEaP* module of AmberTools14. The proteins were successively embedded in 1-palmitoyl-2-oleoyl-sn-glycero-3-phosphoethanolamine (POPE) bilayer patches, solvated with explicit TIP3P water model, and neutralized with the required number of randomly placed K^+^ ions^28, 29, 64^. The ions count was suitably adjusted to account for an osmolarity of 0.15 M KCl. Embedding of the protein into a pre-equilibrated POPE bilayer patch was performed using the PPM server^65^ and subsequently the CharmmGUI tool^66^. The lipid residue nomenclature was converted from the CHARMM to AMBER format using the *charmmlipid2amber*.*py* python script provided with AmberTools. The central pore lipids were then added after calculating the number of lipids to be added to each leaflet by dividing the approximate area of the central pore by the standard area per lipid of POPE molecules^67^. Periodic boundary conditions were used and the distance between the protein and the edge of the box was set to be at least 30 Å in each direction.

Multi-step energy minimization with a combination of steepest descent and conjugate gradient methods was carried out using the *pmemd* program implemented in AMBER14 to relax internal constrains of the systems by gradually releasing positional restraints. Following this, the systems were heated from 0 to 310 K by a 1 ns heating (0–100 K) under constant volume (NVT) followed by 5 ns of constant pressure heating (NPT) (100–310 K) with the phosphorous heads of lipids restrained along the *z*-axis to allow membrane merging and to bring the atmospheric pressure of the system to 1 bar. Langevin thermostat (collision frequency of 1 ps^−1^) was used to maintain a constant temperature, and multiple short equilibration steps of 500 ps under anisotropic pressure scaling (Berendsen barostat) in NPT conditions were performed to equilibrate the box dimensions. A time step of 2 fs was used during all these runs, while post-equilibrium MD simulations were carried out with a time step of 4 fs under constant volume conditions after hydrogen mass repartitioning^68^. The particle-mesh Ewald (PME) algorithm was used to evaluate long-range electrostatic forces with a non-bonded cutoff of 9 Å. During the MD simulations, the length of all R–H bonds was constrained with SHAKE algorithm. Coordinates were saved every 100 ps. The ff14SB^69^ version of the all-atom Amber force field was used to represent the protein systems while lipid14^67^ parameters were used for the POPE bilayer. After equilibration, multi-copy µs-long MD simulations were performed for each system, namely two ~3 μs-long production simulations for each transporter (for a total simulation time of ~12 μs). Trajectory analysis was done using *cpptraj* module of AmberTools14 and VMD1.9.1, and graphs were plotted using the *xmgrace* tool.

### Principal component analysis

To characterize and highlight possible similarities and differences in the collective motions of the binding pockets, we calculated the covariance matrices from the equilibrium trajectory and performed a principal component analysis^70, 71^. As customary in principal component analysis, the covariance matrix was constructed taking the three-dimensional positional fluctuations of Cα atoms from their ensemble average position (after least-squares fitting to remove rotational and translational motion). Diagonalization of the covariance matrix yields a set of eigenvectors and corresponding eigenvalues, which represent the direction and amplitude of the motion, respectively. The eigenvectors are then ranked according to the decreasing order of their associated eigenvalues, such that the first eigenvector represents the largest contribution to the total fluctuation of the system. To visualize the motions represented by the eigenvectors, the structures from the trajectories can be projected onto each eigenvector of interest [principal component (PC)] and transformed back into Cartesian coordinates. The two extreme projections along each eigenvector can then be interpolated to create an animation or compared to understand which parts of the protein are moving according to that specific eigenvector and to what extent. Usually, (a combination of) the first few principal components are able to represent most of the collective motions (the “essential dynamics”^70^) occurring in an MD simulation among the different regions of a protein.

### Clustering of MD trajectories

A cluster analysis of the MD trajectories was performed using the average-linkage hierarchical agglomerative clustering method implemented in *cpptraj* module of AMBER. Such clustering helps to reduce the number of structures for analysis yet retaining the large conformational space sampled during the MD runs. In this approach, we clustered in two separate instances the trajectory based on root mean square deviation (RMSD) (cutoff set to 3 Å) of the AP in the *Loose* protomer and of the DP in the *Tight* protomer. For each protein, the representative structures from each of the 10 top clusters generated in each of the two cases considered (AP in *Loose*, DP in *Tight*) were used to perform quantitative analyses in order to account for dynamical behavior. Except for hydration analyses, all non-protein molecules were stripped from the trajectory during post-processing to reduce additional memory usage and to speed up file processing.

### Pocket descriptors

The list of the pocket descriptors identified for the present study includes: i) cavity volume and shape; ii) molecular lipophilicity potential; iii) electrostatic potential; iv) site hydration; v) fragment-based binding site characterization. The various pocket descriptors used to characterize the binding site were calculated using specific programs after validating their applicability to RND systems by assessing results against available crystal structures and experimental data, as well as previous computational reports^29, 30, 33, 35, 45, 64^.

#### Cavity volume and shape

Evolution of size and shape of the AP and DP during the MD simulations was examined using the two-probe sphere method of *rbcavity* program bundled in the rDock suite^72^. This allows obtaining detailed information on the pocket volume and plasticity of the site. In this method, the binding site volume was identified by a fast grid-based cavity detection algorithm^73^ within a sphere of radius 14 Å, centred over the pockets, using large and small probe radii of 6.0 Å and 1.5 Å, respectively. These radii were found to be optimal for our case after evaluating different combinations and checking through visual inspection their accuracy in predicting volume of the pocket space by keeping the possible inclusion of regions extending outside the pocket of interest at its least. rDock also gives information about the approximate shape of the pocket; we could thus provide approximate values for the minimal cross-sectional area associated to each cavity.

#### Molecular lipophilicity potential

The three-dimensional distribution of lipophilicity in space or on a molecular surface can be described using Molecular Lipophilicity Potential (MLP), which represents the influence of all lipophilic fragmental contributions of a molecule on its environment. The MLP value of a point in space (*k*) is generated as the result of intermolecular interactions between all fragments in the molecule and the solvent system, at that given point. Thus, MLP can be calculated from the fragmental system of logP and a distance function as shown in the following equation^74^:

1 $$ML{P}_{k}=\sum _{i=1}^{N}{F}_{i}.f({d}_{ik})$$

where *N* is the number of fragments, *F*_*i*_ is the lipophilic contribution of fragment *i* of the molecule and *f*(*d*_*ik*_) is a function based on the distance of the measured point in space *k* to fragment *i*.

In this way, summing up all positive and all negative MLP values associated to each point on the binding pocket yields the lipophilic index (LI) as:

2 $$LI=\frac{{\rm{\Sigma }}ML{P}^{+}}{{\rm{\Sigma }}ML{P}^{+}+|{\rm{\Sigma }}ML{P}^{-}|\,}.100$$

The lipophilicity of AP in the *Loose* protomer and DP in the *Tight* protomer were qualitatively and quantitatively estimated in this way using MLP Tools^75^ plugin available for PyMOL.

#### Electrostatic potential

The electrostatic potential surface maps were computed by APBS^76^, after preprocessing structures of AcrB and AcrD to assign charges and atomic radii using the PDB2PQR server^77^. All electrostatic potential calculations were performed at 0.15 M physiological salt concentration, with a solvent probe of radius 1.4 Å, a solvent dielectric constant of 78.5, a biomolecular dielectric constant of 2.0, a temperature of 310 K, a minimum grid spacing of 0.5 Å and keeping the other Poisson-Boltzmann parameters at default.

#### Hydration analysis

The radial distribution function (RDF) indicates the probability of finding water molecules at a certain distance from a region or residue of interest and is commonly used to analyse the solution structure revealed from either experimental or computer simulations data.

The RDF analysis of water oxygen atoms was performed using *cpptraj* module of AMBER14, in which the RDF is computed from the histogram of the number of solvent particles found as a function of the distance *R* from an (ensemble of) atom(s), normalized by the expected number of solvent particles at that distance in bulk. The normalization is estimated from:

3 $$Density\,\ast \,([\frac{4\pi }{3}{(R+dR)}^{3}]-\,[\frac{4\pi }{3}d{R}^{3}])$$

where *dR* is equal to the bin spacing, the default *density* value is 0.033456 molecules Å^−3^, which corresponds to density of water approximately equal to 1.0 g mL^−1^. Bin spacing of 0.1 and a maximum bin value of 4.0 was used in this case to calculate the RDF of all water oxygen atoms to each atom of AP in the *Loose* protomer and of DP in the *Tight* protomer over the entire length of the simulation.

Though RDF clearly shows a difference in the water distribution around the desired regions, it lacks the ability to present the information about the spatial positions of these differences. Hence, spatial distribution function (SDF) of waters around the whole protein was calculated using the Gromacs utility *g_spatial* ^78^ on the trajectory frames grouped into the most populated conformational clusters extracted from MD simulations. SDF allows to determine the three-dimensional density distribution of aqueous solution around the binding pockets of the transporters. Density isovalue gives information regarding the relative number densities with respect to the average number density of solvent molecules in bulk. RDF and SDF together highlight the hydration around the binding pockets of these proteins, which can be effectively used to understand the molecular mechanism of interaction of water molecules penetrating the pocket in a dynamic manner.

#### Fragment-Based Binding Site Characterization

The FTMap server^79^ implementing the FTSite algorithm is a tool helpful in the identification of binding sites and of the fragments that could be possible source of structure- and fragment-based drug design attempts. The main aim of such fragment-based binding site analysis is to obtain a measure of the ability of the protein (and in particular the pockets under study) to bind a drug-like molecule.

FTMap identifies the important hot spots based on the consensus clusters of 16 standard probes which include molecules varying in size, shape and polarity (Supplementary Fig. S11). Such a diverse library of probes is useful to capture a range of interaction types that include hydrophilic, hydrophobic, hydrogen-bonding and aromatic interactions. The regions where clusters of different probes of the same or different type overlap are marked as consensus (CS) and multi-functional (MFS) sites, respectively, and are ranked based on the number of their clusters. Clusters in close proximity to a top ranked cluster are merged with it and the protein residues within this region become the top ranked putative ligand binding site.

## Electronic supplementary material

Supplementary Information
