# Supplementary material for: Molecular Rationale behind the Differential Substrate Specificity of Bacterial RND Multi-Drug Transporters
Source: Sci Rep. 2017 Aug 14;7:8075. doi: 10.1038/s41598-017-08747-8 (PMC5556075; doi:10.1038/s41598-017-08747-8)
Supplement: Supplementary file 1 — Supplementary Information [file 41598_2017_8747_MOESM1_ESM.pdf]

Supplementary Information

Molecular Rationale behind the Differential Substrate Specificity of Bacterial RND Multi-Drug Transporters

Venkata Krishnan Ramaswamy[1], Attilio V. Vargiu[1], Giuliano Malloci[1], Jürg Dreier[2], Paolo Ruggerone[1*]

[1] Department of Physics, University of Cagliari, Cittadella Universitaria, S.P. Monserrato-Sestu km 0.700, I-09042 Monserrato (CA), Italy
[2] Basilea Pharmaceutica International Ltd., Grenzacherstrasse 487, 4058 Basel, Switzerland
* Corresponding author: paolo.ruggerone@dsf.unica.it

Homology modeling of AcrD

Visual inspection of the top 5 homology models generated by Modeller revealed an identical overall 3-dimensional structural fold with minor difference in the loop regions. The final AcrD model featured 97.3% and 99.7% of the residues in the favored and allowed regions of the Ramachandran plot, respectively. The evaluation results were fully within the permissible limits of a good model and none of the outlier residues (10 of 3105 accounting to 0.3%) were in the regions described in this study. The overall quality factor for non-bonded atomic interactions assessed with ERRAT plot further confirmed the good quality of model with a score of 97.2%, which is in the range of high-resolution experimental structures. The ProSA evaluation showed that the overall model quality score (Z-score) of the homology model (-12.2) and the template crystal structure (-12.6) were almost similar and within the range of scores typically found for native proteins of similar size. The local quality of the model based on interaction energies for each residue as evaluated with ProSA served as additional proof of model quality with most of the residues falling in the negative energy scale and only a minor set on the positive side. All the observed values were within ranges also observed for the experimentally (X-ray) determined template structure (PDB ID: 4DX5), increasing the confidence in the AcrD homology model obtained.

The evaluation results are summarized in Table S1, which also shows superimposition of the AcrD homology model with the AcrB template structure (PDB ID: 4DX5), highlighting the similarity in their general structural fold.

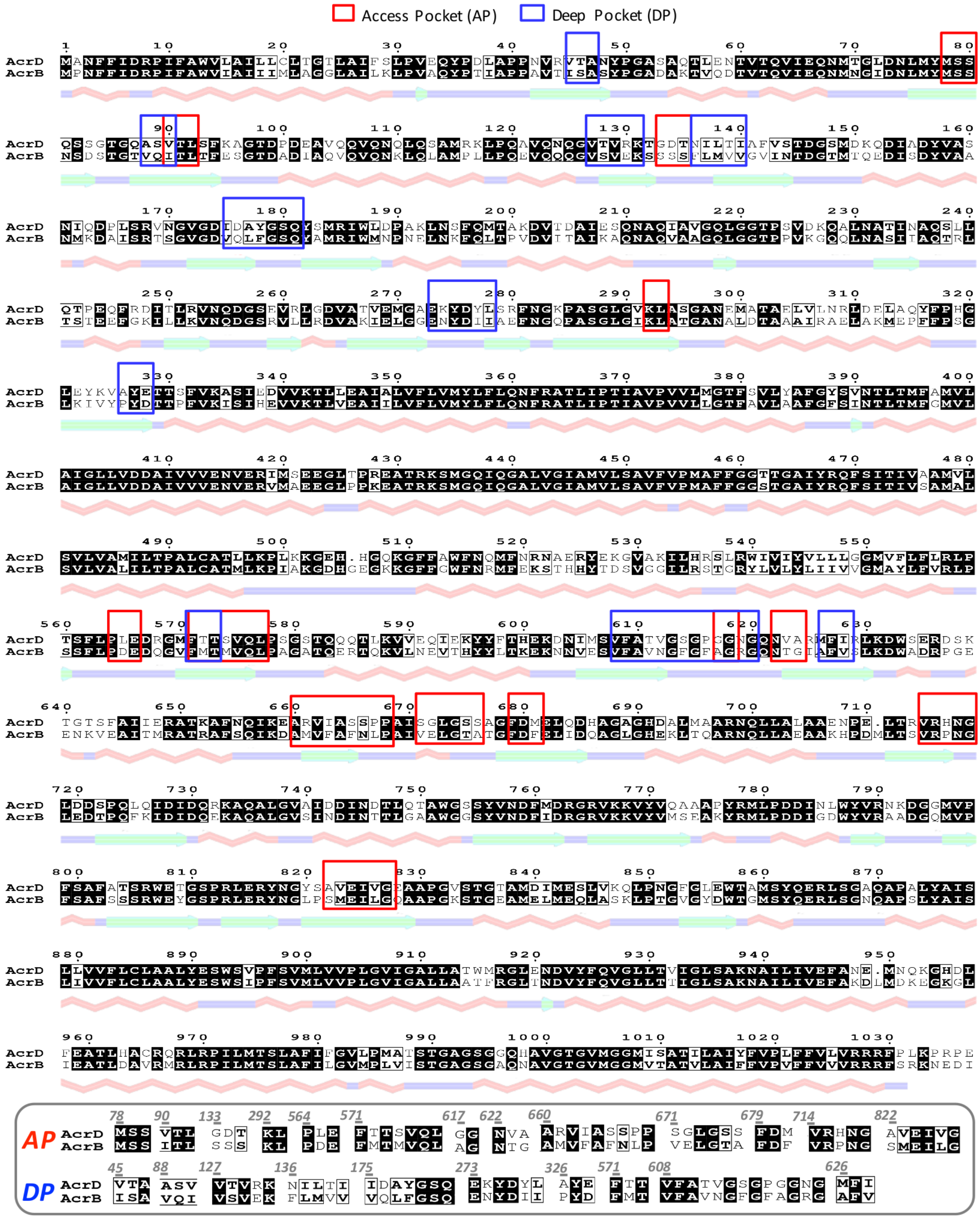

Figure S1. Sequence alignment of AcrD with AcrB. *The regions corresponding to Access Pocket (AP) and Deep Pocket (DP) are marked in red and blue boxes, respectively. The secondary structure of the sequence is also shown with β-sheet in green, α-helix in red and coil in blue. Identical residues are highlighted with black filled boxes, similar with color less boxes while all others are mismatches*

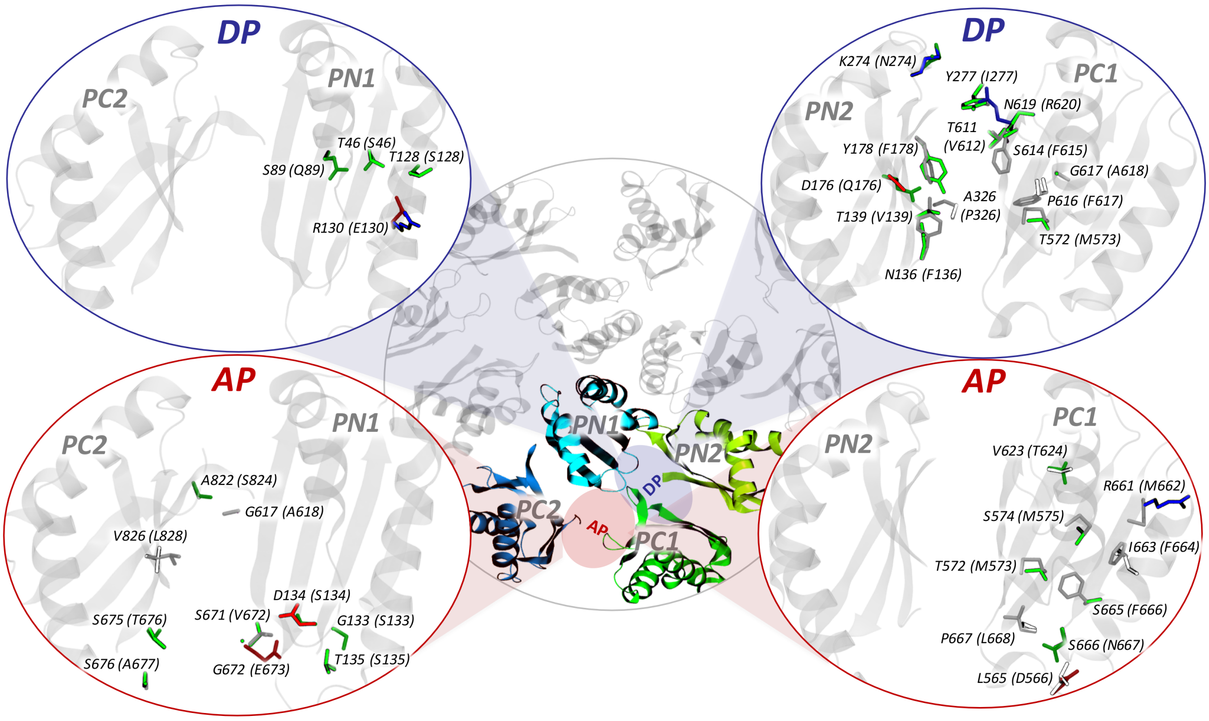

Figure S2. Sequence comparison of binding pockets (AP and DP) between AcrB and AcrD mapped onto their structures. *The figure in the middle shows the four main domains (colored differently) enclosing the AP and DP. The locations of the pockets are schematically shown as red and blue colored circles for AP and DP, respectively. The insets highlight the mismatched residues of AcrB (BrushedMetal) and AcrD (Goodsell) as licorice colored by residue type (non-polar residues in white, polar residues in green, basic residues in blue and acidic residues in red). The residue labelling follows the notation 'AcrD (AcrB)'.*

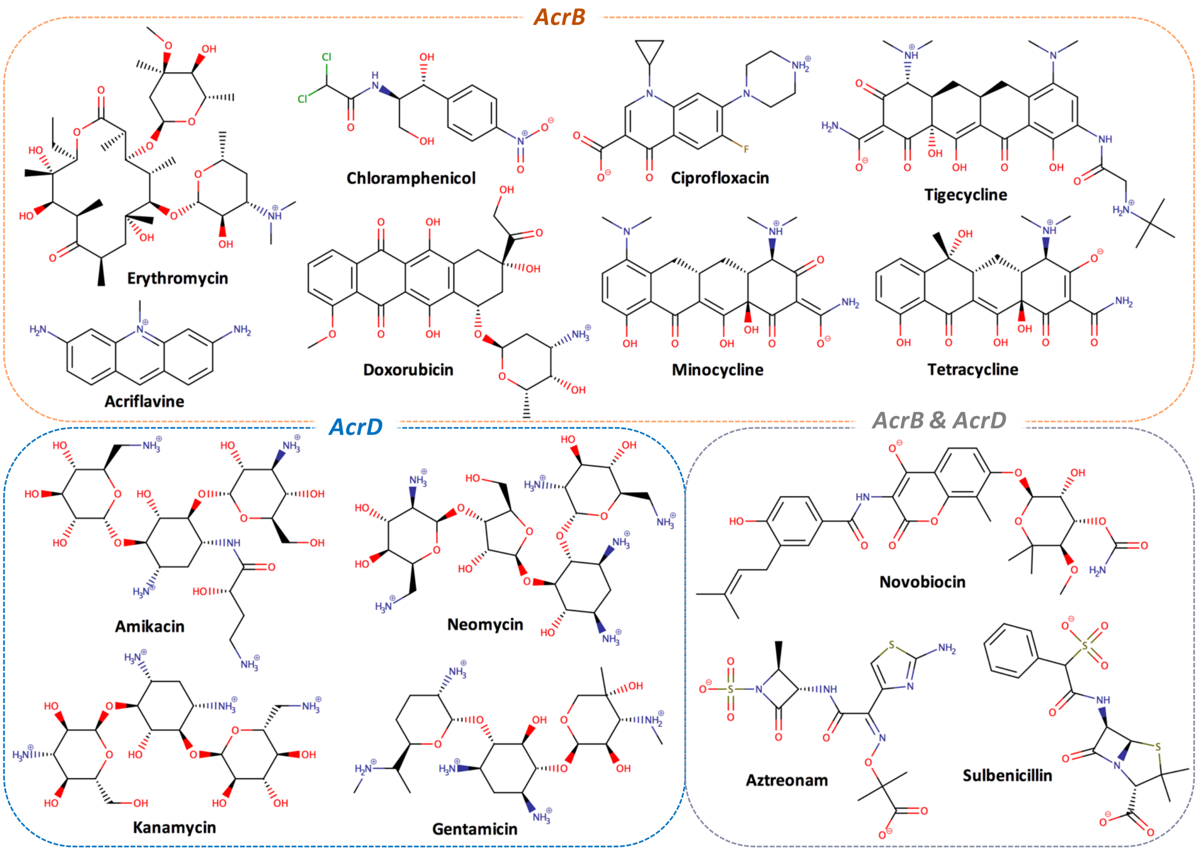

Figure S3. Examples of antibiotic substrates of AcrB and AcrD in E. coli. *Orange and blue frames indicate substrates of AcrB and AcrD, respectively, while the grey frame indicates the substrates common to both AcrB and AcrD.*

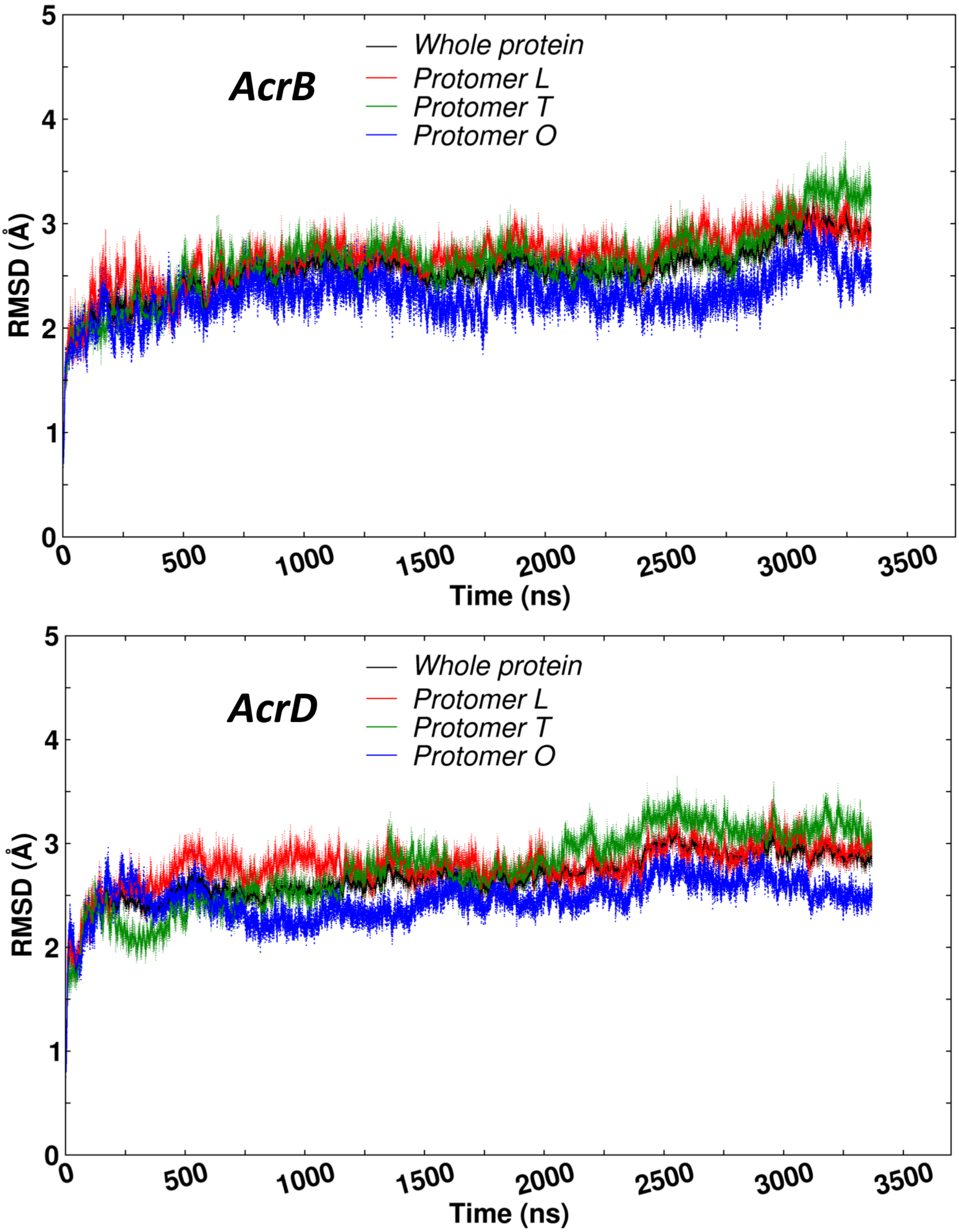

Figure S4. RMSD of the backbone of whole proteins and of each protomer for AcrB (upper panel) and AcrD (lower panel). *The abbreviations L, T and O in the figure legends refer to Loose, Tight and Open conformational states of the protomer.*

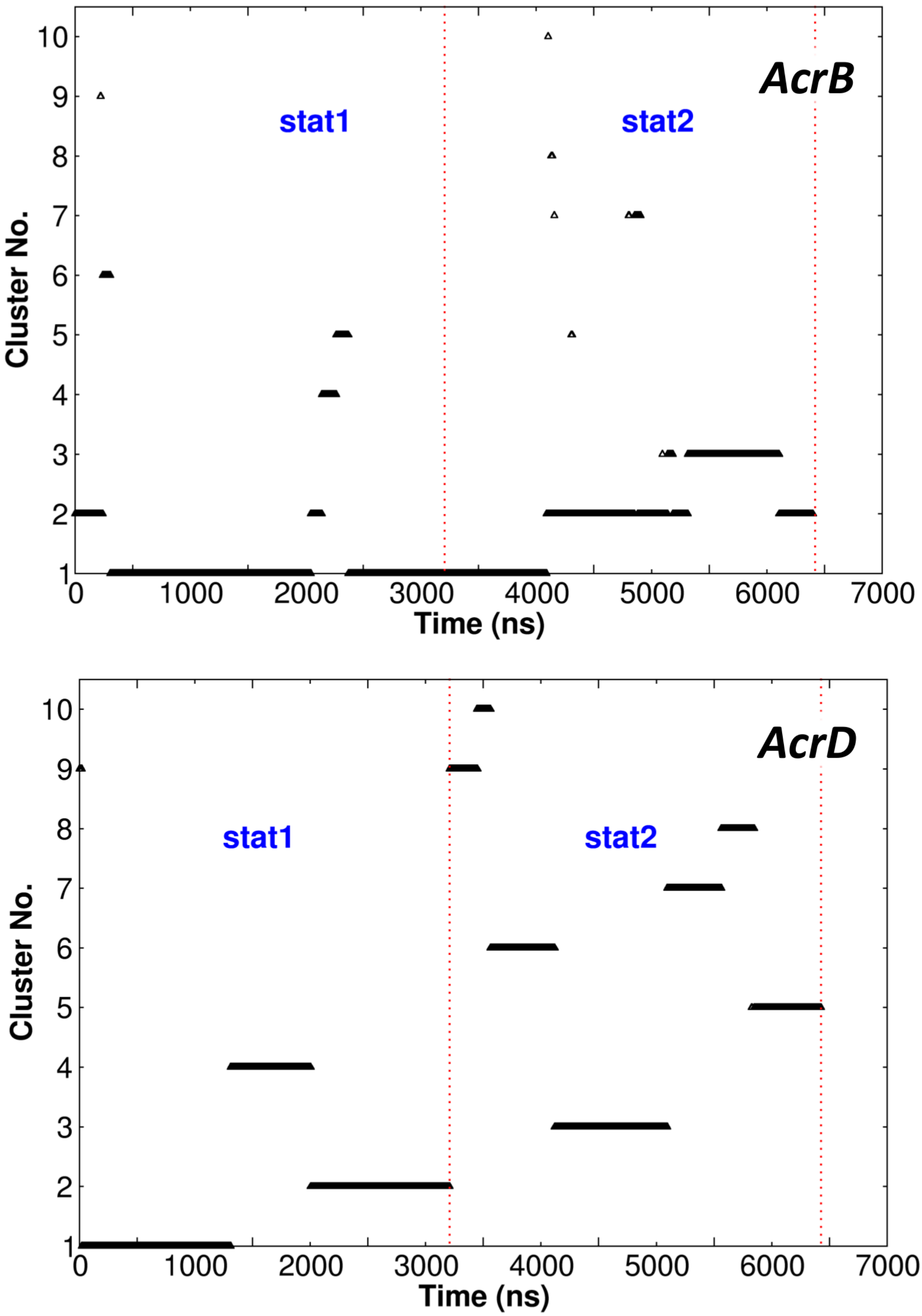

Figure S5. Cluster evolution timeline for AP in the Loose protomer of AcrB (upper panel) and AcrD (lower panel). *The red dotted lines mark the corresponding trajectory sections of the statistical runs (stat1 and stat2).*

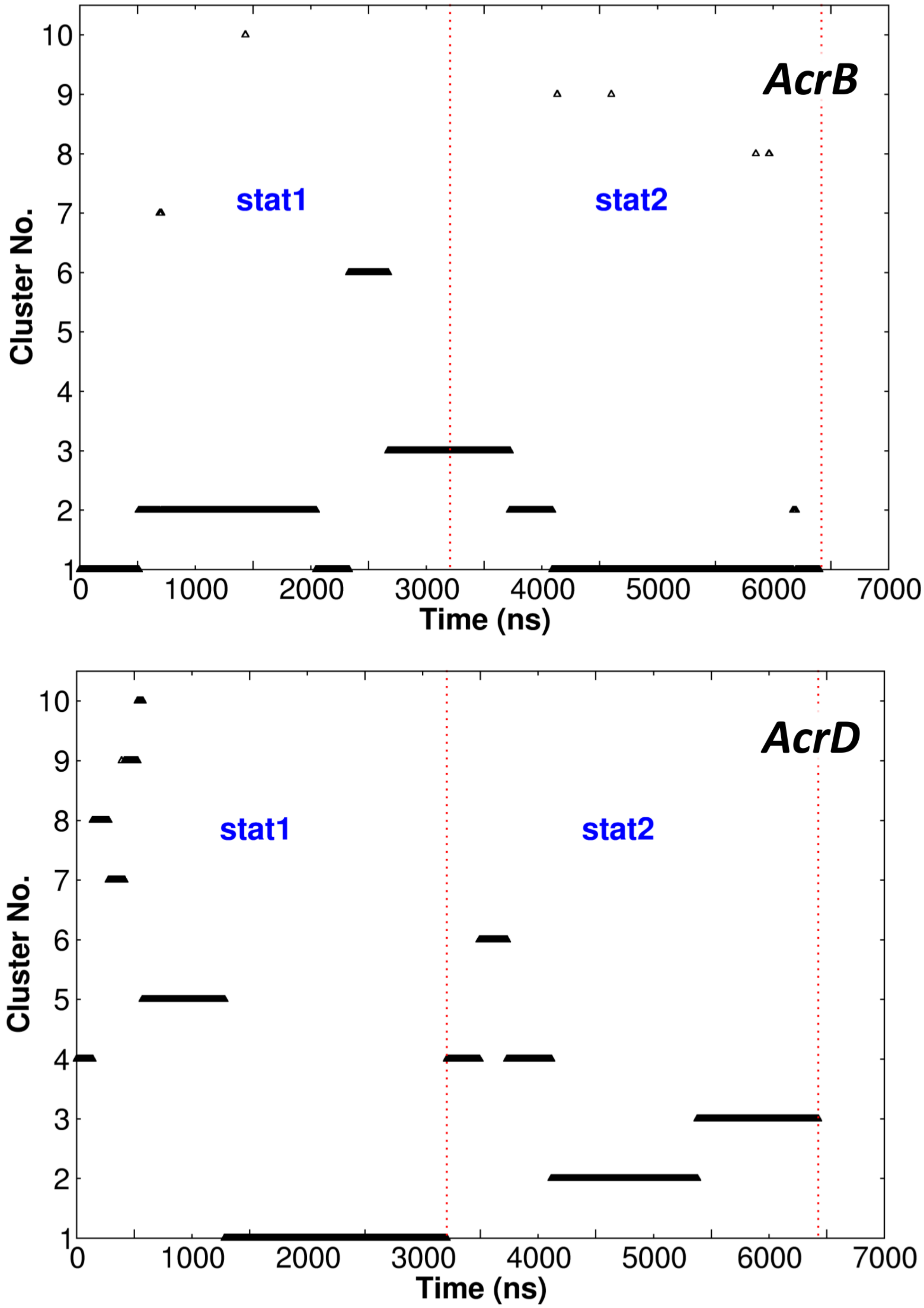

Figure S6. Cluster evolution timeline for DP in the Tight protomer of AcrB (upper panel) and AcrD (lower panel). *The red dotted lines mark the corresponding trajectory sections of the statistical runs (stat1 and stat2).*

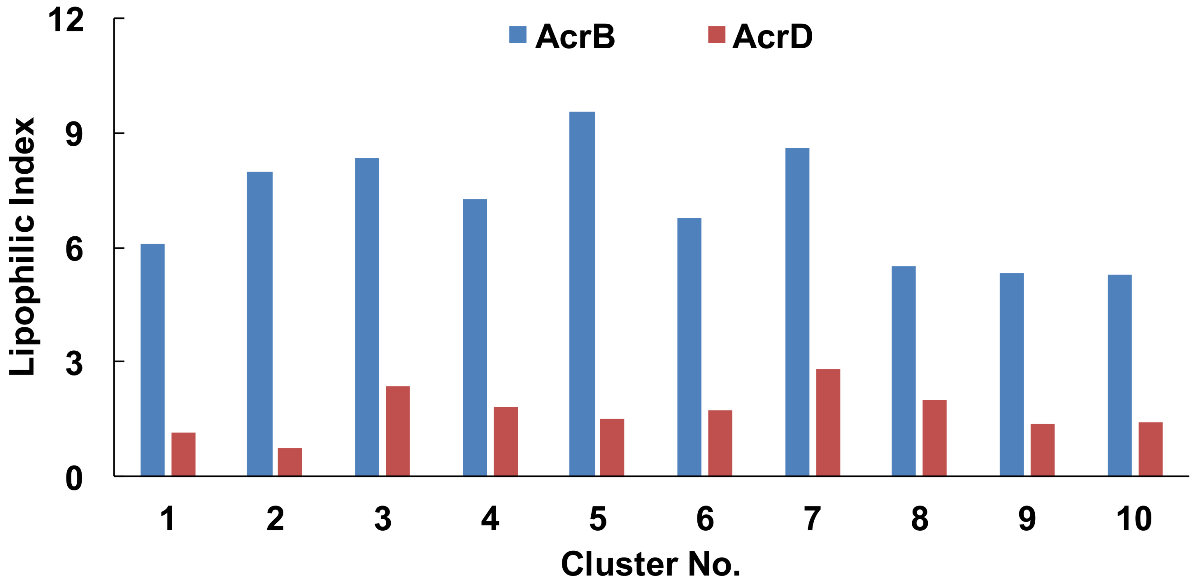

Figure S7. Distribution of the lipophilic index for AP in the Loose protomer of AcrB and AcrD over the clusters.

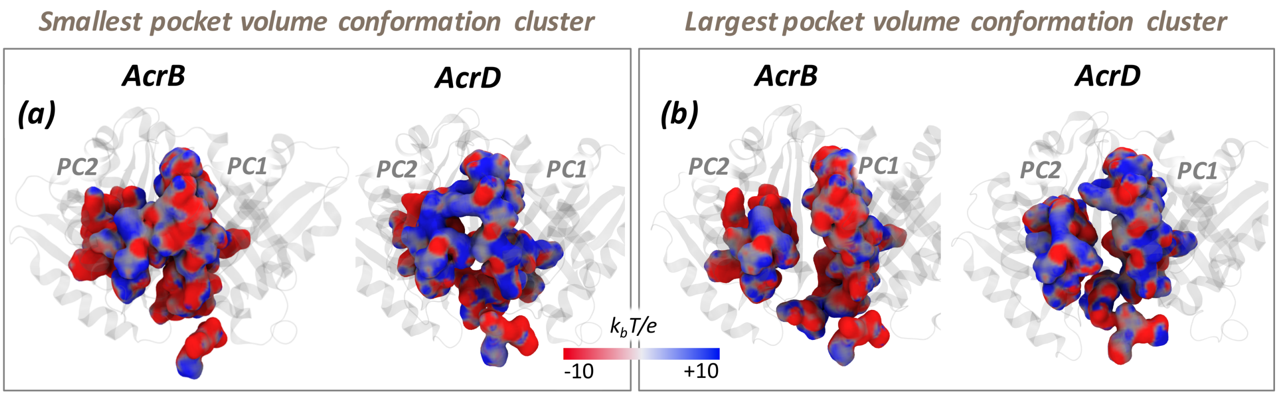

Figure S8. Electrostatic potential plotted on the molecular surface representation of AP in the Loose protomer of AcrB and AcrD for the cluster conformations with (a) smallest and (b) largest pocket volumes sampled during the MD simulations. *See Figure 4 in the main text for further details.*

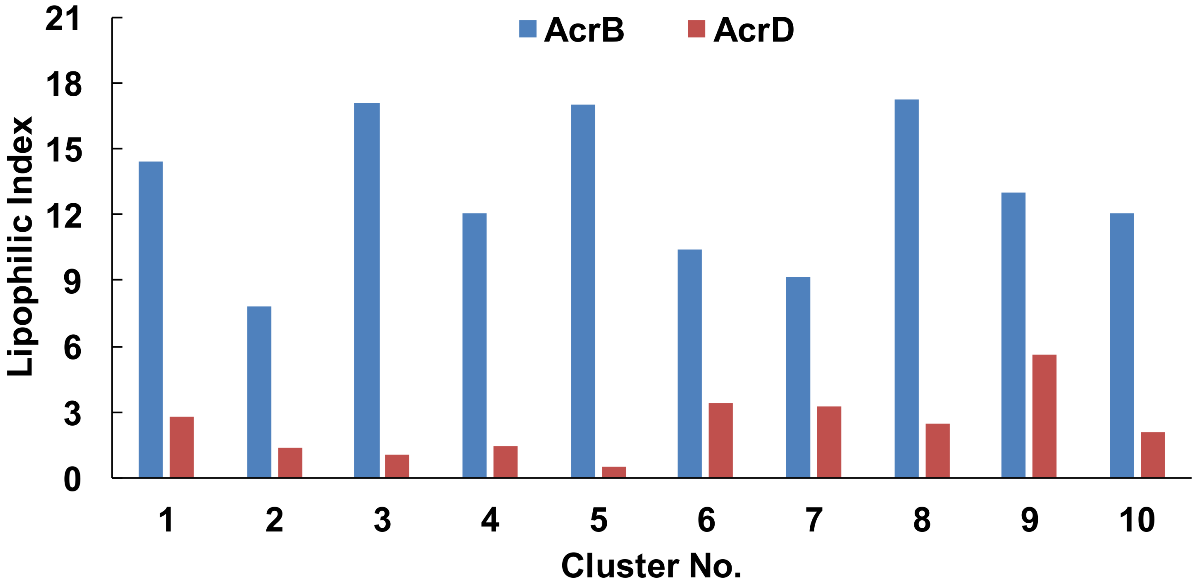

Figure S9. Distribution of the lipophilic index for DP in the Tight protomer of AcrB and AcrD over the clusters.

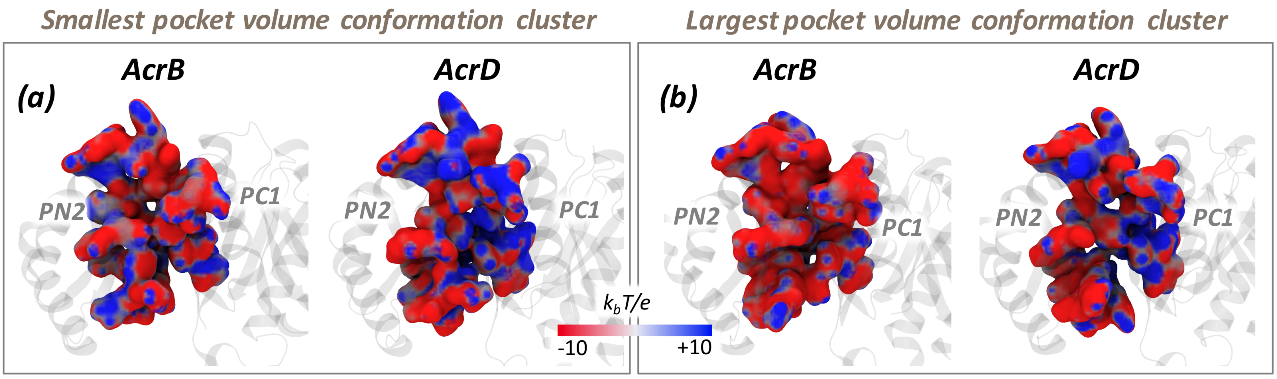

Figure S10. Electrostatic potential plotted on the molecular surface representation of DP in the Tight protomer of AcrB and AcrD for the cluster conformations with (a) smallest and (b) largest pocket volumes sampled during the MD simulations. *See Figure 4 in the main text for further details.*

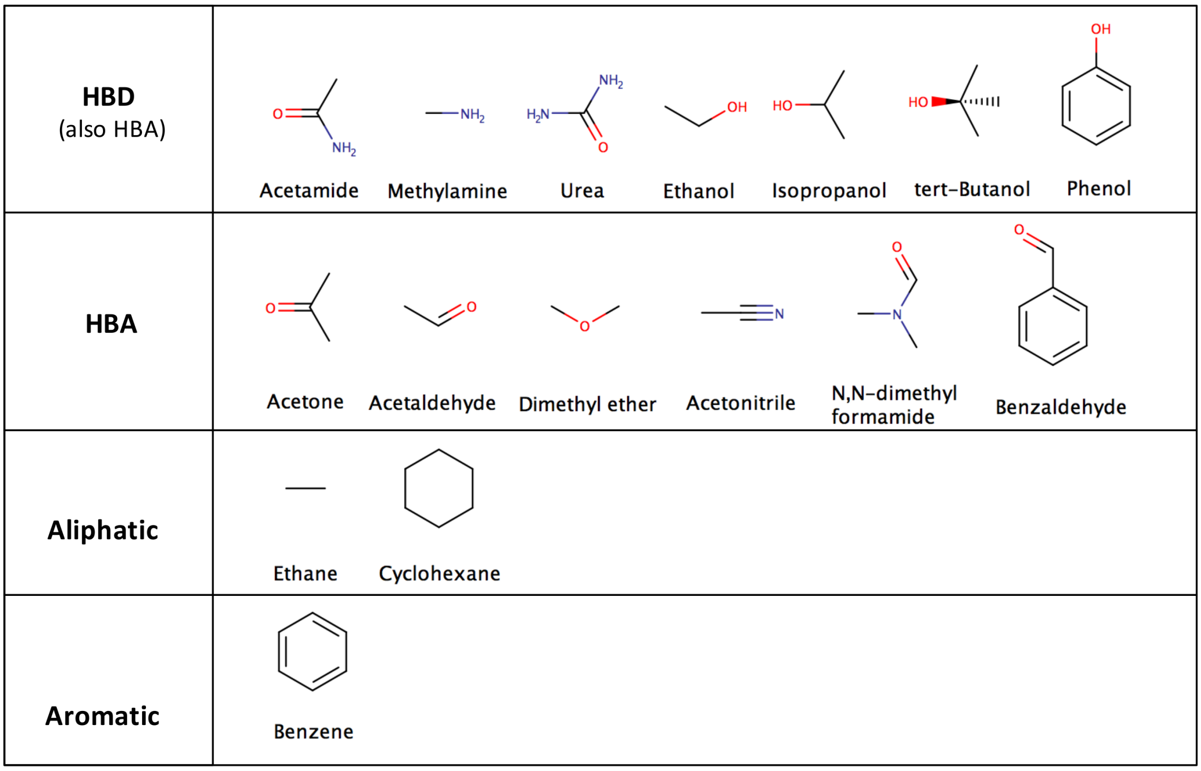

Figure S11. The standard repertoire of small organic probe molecules used by FTMap. *(HBD: Hydrogen Bond Donors; HBA: Hydrogen Bond Acceptors)*

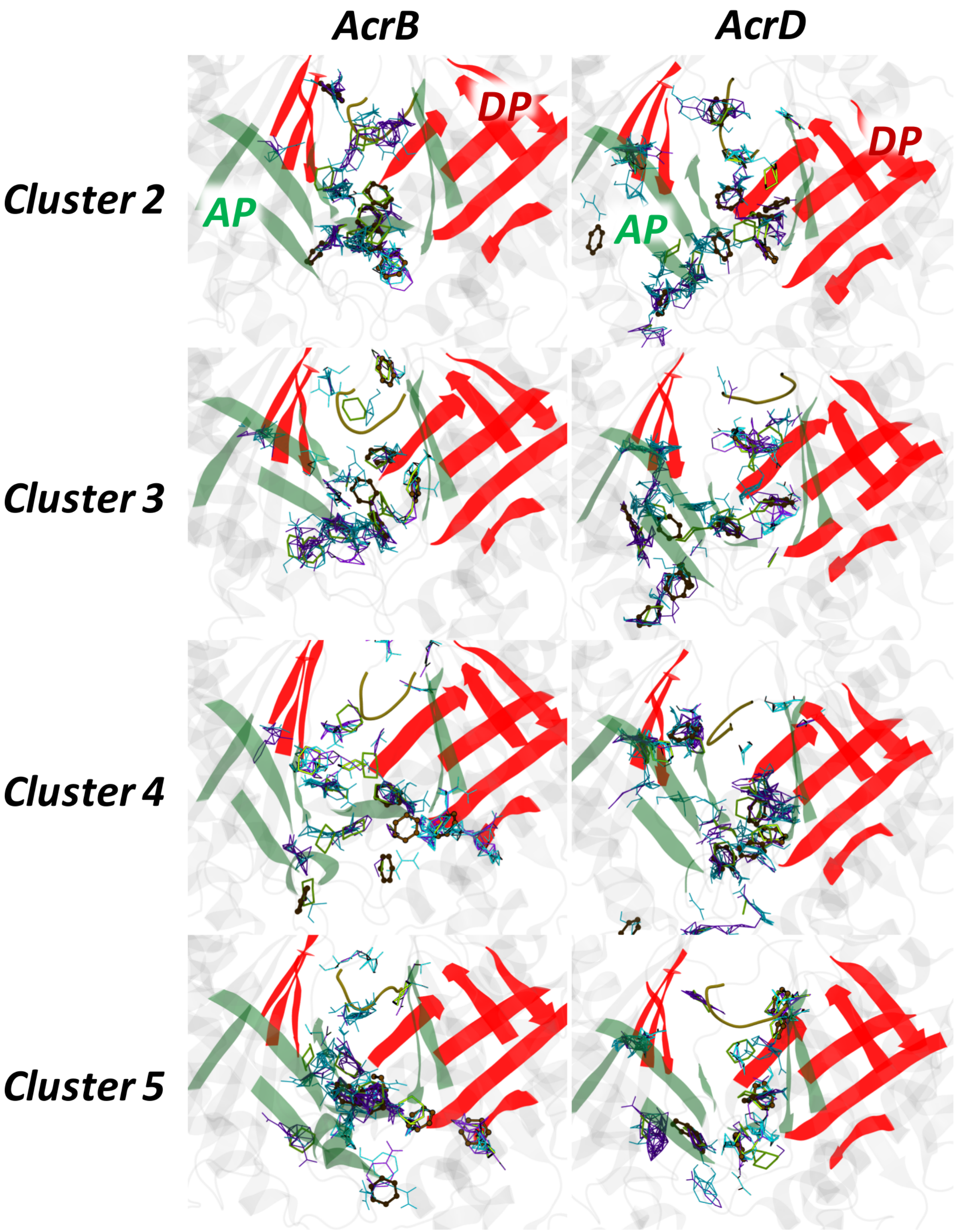

Figure S12. The distribution of various MFSs within the AP and DP of top five cluster representatives. *See Fig. 9 in main text for cluster 1. The binding modes of the different probes are shown as lines for hydrogen-bond donor (cyan), hydrogen-bond acceptor (violet) and aliphatic (yellow), and as CPK for aromatic (ochre) ligands. The AP and DP are marked in green and red, respectively, while the G-loop in yellow cartoon representations. (Note: The categorizing of MFSs here is arbitrary due to indistinct boundaries between the pockets)*

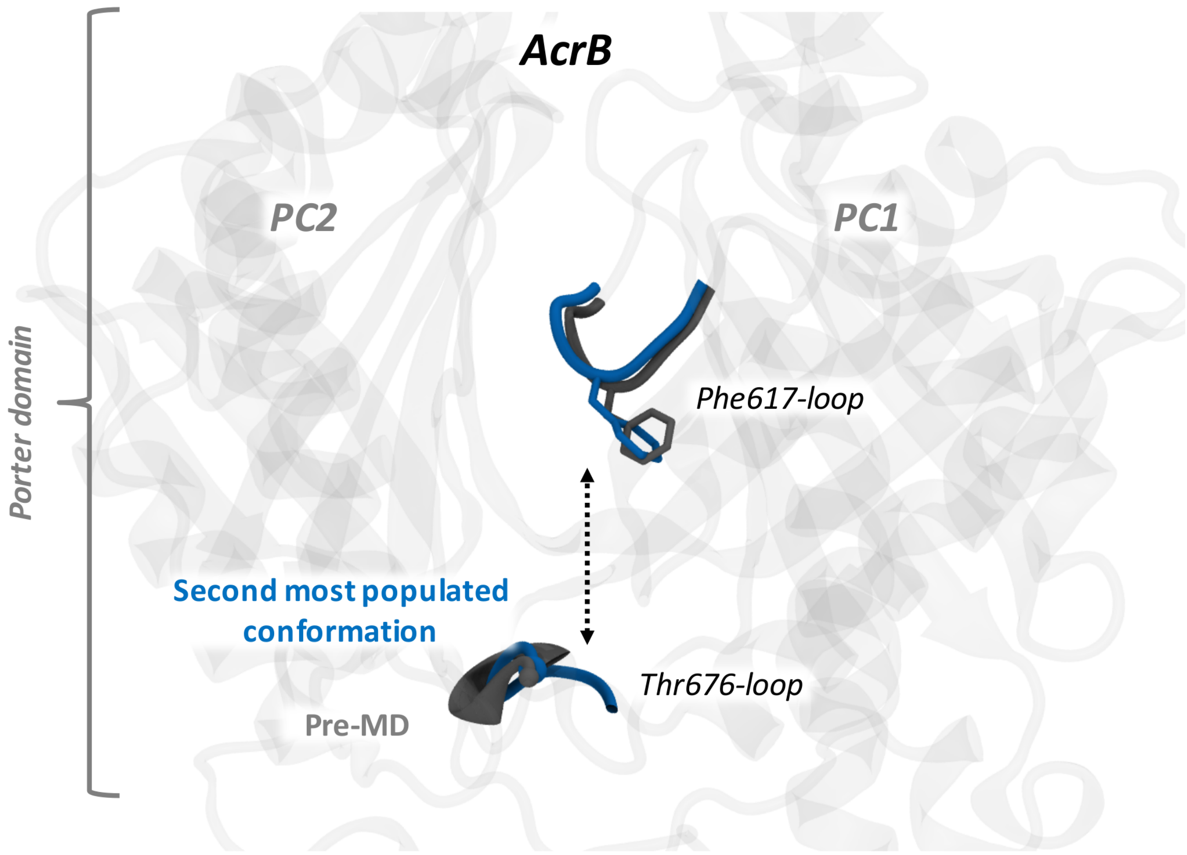

Figure S13. The conformational states of the bottom-loop (Thr676-loop) in the Loose protomer of the second most populated cluster (shown in blue) and the pre-MD (shown in grey) configurations of AcrB. *The conformations of the G-loop (Phe617-loop) are also reported.*

Table S1. Evaluation results of the AcrB-based AcrD model. *The figure on the left shows the structural superposition of AcrD (colored representation) onto AcrB (gray representation). Only a single protomer is shown for the sake of clarity. The table on the right shows the model evaluation results.*

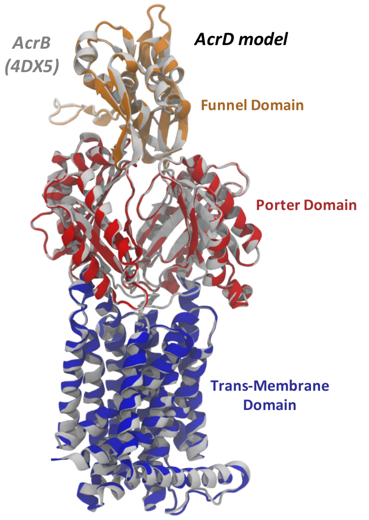

Evaluation Criteria	AcrB-based AcrD model
RMSD (backbone)	0.12 Å
TM-score	0.99
Ramachandran favoured	97.33%
ERRAT	97.21%
Verify-3D	Pass

Table S2. Volume and minimal projection area of AP in the Loose protomer of AcrB and AcrD. *For AcrB, the pre-MD structure corresponds to the crystal structure identified by PDB code 4DX5 while for AcrD it is the final optimized homology model used as starting configuration for MD simulations.*

**System**	**Pre-MD**	**MD clusters**
	**Volume (Å^3)**	
AcrB	3620	2515 ± 438
AcrD	3760	3015 ± 385
	**Minimal Projection Area (Å^2)**	
AcrB	600	543 ± 25
AcrD	650	580 ± 52

Note 1: The volume of AP in the Loose protomer considering a set of 10 different AcrB crystal structures (PDB codes: 2DHH, 2GIF, 2J8S, 3W9H, 4DX5, 4DX7, 4U8V, 4U8Y, 4U95, 4U96) was 3740±143 Å^3 with a minimal projection area of 586±28 Å^2.

Note 2: The minimal projection area of the largest antibiotic substrates of these transporters are 106 Å^2 (erythromycin for AcrB) and 81 Å^2 (neomycin for AcrD)[1].

Table S3. Volume and minimal projection area of DP in the Tight protomer of AcrB and AcrD. *See Table S2 for further details.*

System	Pre-MD	MD clusters
**Volume (Å^3)**		
AcrB	3708	2610 ± 250
AcrD	3855	2770 ± 306
**Minimal Projection Area (Å^2)**		
AcrB	506	491 ± 30
AcrD	586	500 ± 67

Note: The volume of DP in the Tight protomer considering a set of 10 different AcrB crystal structures was 3761±93 Å^3 with a minimal projection area of 567±44 Å^2.

Table S4. Volumes and minimal projection areas of DP. *Volumes and minimal projection areas of DP in the Tight protomer of AcrB crystal structures and structures extracted from simulations of AcrB in the presence of benzene [unpublished study]. See Table S2 for further details.*

**AcrB System**	**Volume (Å^3)**	**Minimal Projection Area (Å^2)**
Crystal Structures	3761 ± 93	567 ± 44
Benzene bound complexes	3406 ± 207	549 ± 57

Supplementary References:

1. Malloci, G. *et al.* A database of force-field parameters, dynamics, and properties of antimicrobial compounds. *Molecules* **20**, 13997-14021, doi:10.3390/molecules200813997 (2015).
